# Focal adhesions Kindlin‐1 and Kindlin‐2 affected by epigenetic modifying in hepatocarcinogenesis

**DOI:** 10.1002/ctm2.1517

**Published:** 2024-01-10

**Authors:** Yang Yuan, Xiaona Dong, Shengju Yin, Guoliang Zhang, Haiyan Zhou, Guohui Li, Yan Tang, Xiaofan Wei, Hongquan Zhang

**Affiliations:** ^1^ Department of Human Anatomy, Histology, and Embryology School of Basic Medical Sciences, Peking University Health Science Center Beijing China; ^2^ Department of Pharmacology School of Basic Medical Sciences, Peking University Health Science Center Beijing China; ^3^ Department of Pharmacy National Cancer Center/National Clinical Research Center for Cancer/Cancer Hospital, Chinese Academy of Medical Sciences and Peking Union Medical College Beijing China

Dear Editor,

Hepatocarcinogenesis is significantly different among ethnic groups, which is higher in Chinese than Caucasian and African populations, but the exact mechanism is unclear (https://www.thno.org/v13p1607.htm).^1^ Focal adhesions Kindlin‐1 and Kindlin‐2 are integrin‐interacting proteins that activate transmembrane receptor integrin and regulate tumour cell growth, invasion and metastasis.^2^, ^3^, ^4^ This study aims to investigate whether genetic and epigenetic factors^5^ of Kindlin‐1 and Kindlin‐2 affect the hepatoma phenotype, so as to provide evidence for the development of potential therapeutic targets for hepatocellular carcinoma.

The distribution frequency of single nucleotide polymorphisms (SNPs) can be used to evaluate genetic variation in a population.^6^ We first analysed the allelic frequency of Kindlin‐1 gene in three functional SNPs including Exon 7 (C>T, rs202037230) (Figure 1A), Exon 11 (C>T, rs2232074) (Figure 1B) and Exon 3 (A>G, rs16991866) (Figure 1C) in Chinese and compared with the reported Caucasian and African populations (Table S1).^1^, ^2^ Subsequently, we analysed the allelic frequencies of Kindlin‐2 gene in three functional SNPs including Exon 11 (C>T, rs777658527) (Figure 1D), Exon 7 (G>A, rs2357947) (Figure 1E) and Exon 4 (T>A/T>C/T>G, rs62003529) (Figure 1F) in Chinese and compared with the reported Caucasian and African populations (Table S2).

**FIGURE 1:**
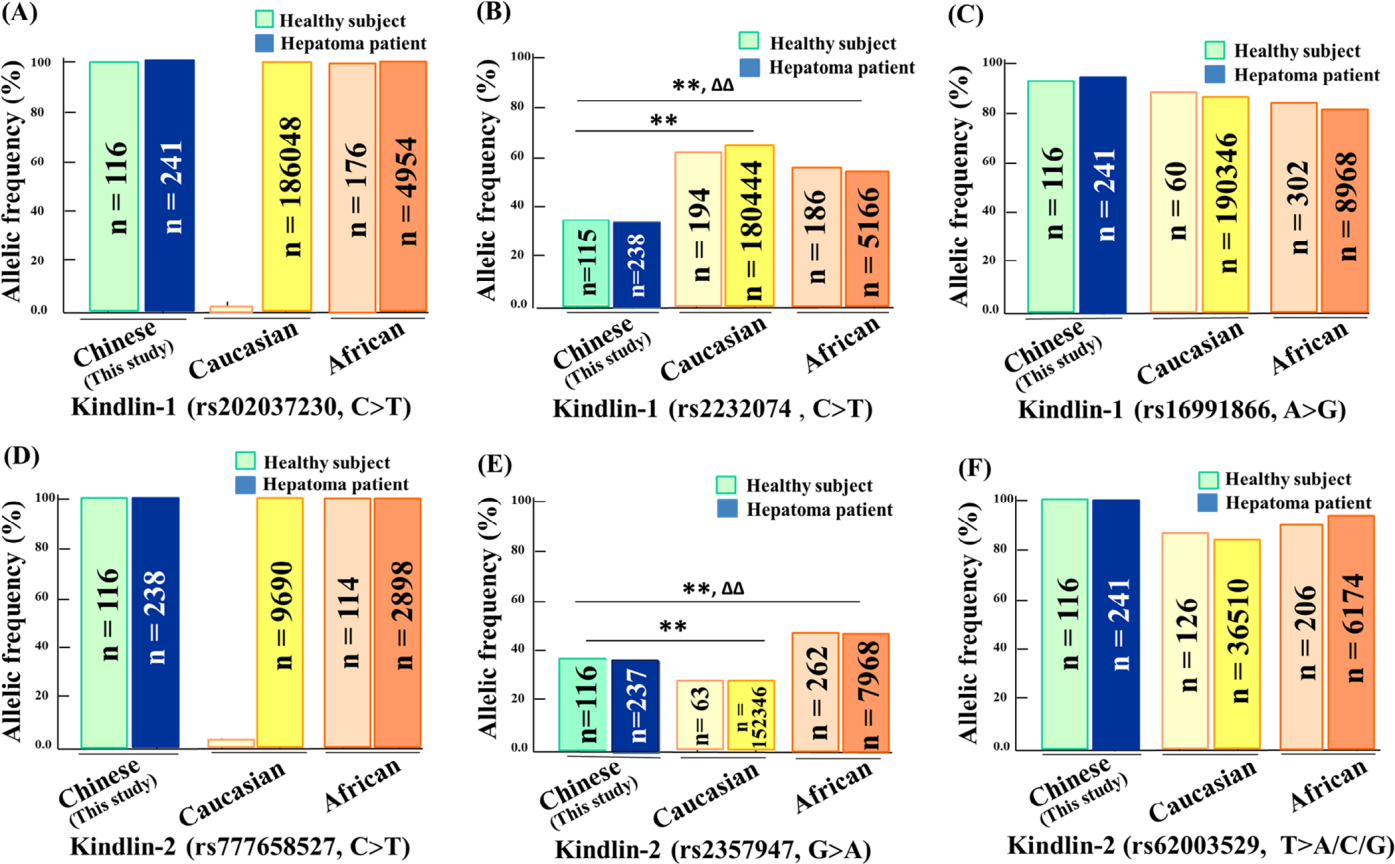
Genetic polymorphisms of focal adhesions (Kindlin‐1 and Kindlin‐2) genes. (A–C) Allelic frequencies of Kindlin‐1 gene in three single nucleotide polymorphisms (SNPs), including (A) Exon 7 (C>T, rs202037230), (B) Exon 11 (C>T, rs2232074) and (C) Exon 3 (A>G, rs16991866), in Chinese healthy subjects (*n* = 116) and hepatoma patients (*n* = 241) compared with previous reported Caucasian (*n* = 194 or 180 444, *p* < .01) and African (*n* = 186 or 5166, *p* < .01) populations. (D–F) Allelic frequencies of Kindlin‐2 gene in three SNPs, including (D) Exon 11 (C>T, rs777658527), (E) Exon 7 (G>A, rs2357947) and (F) Exon 4 (T>A/T>C/T>G, rs62003529), in Chinese healthy subjects (*n* = 116) and hepatoma patients (*n* = 238) compared with previous reported Caucasian (*n* = 63 or 152 346, *p* < .01) and African (*n* = 262 or 7968, *p* < .01) populations. All data are shown as mean ± standard deviation (SD). ^*^ *p* < .05, ^**^ *p* < .01, compared with Chinese healthy subject group; Δ*p* < .05, ΔΔ*p* < .01, compared with Caucasian group; NS: no significance.

The present results showed that allelic frequencies of Kindlin‐1 in rs2232074, C>T (Figure 1B) and Kindlin‐2 in rs2357947, G>A (Figure 1E) in Chinese are significantly different from those in Caucasian and African populations (both *p* < .01). These results support the view that there are significant racial and geographic differences in the incidence of liver cancer epidemiology. However, there was no significant difference in the distribution frequencies of Kindlin‐1 and Kindlin‐2 genes between Chinese healthy subjects and hepatoma patients, suggesting that genetic factors might not to involve in the occurrence of hepatoma in the same genetic background population.

Then, the clinical characteristics between Chinese healthy subjects and hepatoma patients were compared (Table S3). Among the hepatoma patients (*n* = 219), hepatitis B virus (HBV)‐infected persons (>500 copies**/**mL) were 89 (40.6%), while non‐HBV‐infected patients were 130 (59.4%), respectively. As shown in Figure 2A (sector graph), among the healthy subjects (*n* = 116), the males were 52 (44.8%) and the females were 64 (55.2%), while among the hepatoma patients (*n* = 241), there were 216 males (89.6%) and 25 females (10.4%), respectively. Alpha fetoprotein (AFP) has been widely reported as a biomarker for the occurrence and progression of hepatocellular carcinoma.^7^ We performed Kindlins genotyping stratification analysis on serum AFP levels in hepatoma patients. The results are shown in Figure 2B (clustering heatmap). In three SNPs of Kindlin‐1 gene (Table S4) and in three SNPs of Kindlin‐2 gene (Table S5), the significant differences of serum AFP level were not observed among genotypes in Chinese hepatoma patients. These results suggested that the genetic variations of focal adhesion Kindlin‐1 and Kindlin‐2 might not be involved in the progression of liver cancer initialed by HBV and AFP in Chinese patients.

**FIGURE 2:**
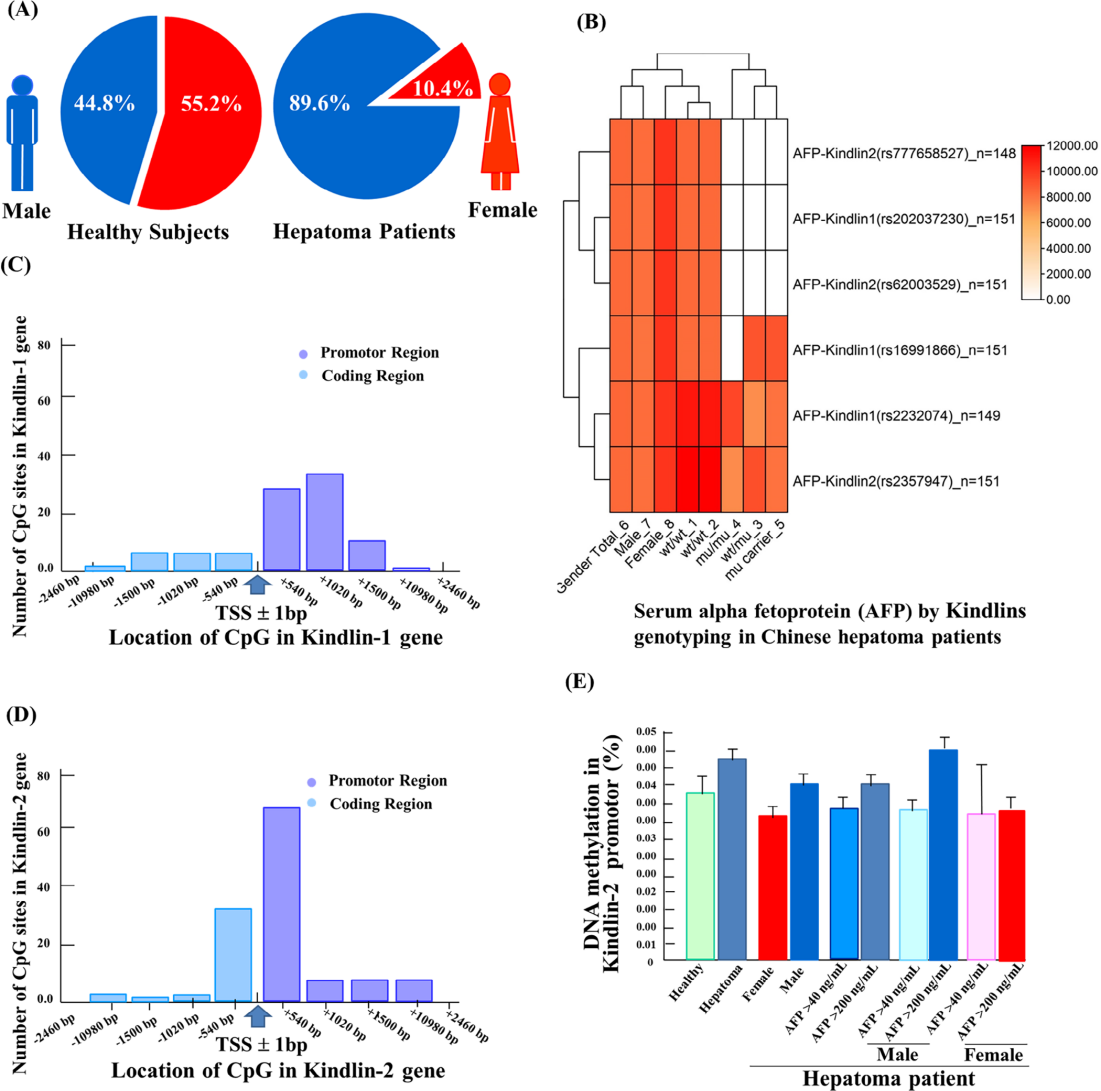
Clinical characteristics of Chinese healthy subjects (*n* = 116) and hepatoma patients (*n* = 241). (A) Sector graph showed that the numbers of male (blue) and female (red) in Chinese healthy subjects (left) and hepatoma patients (right). (B) Cluster heatmap showed that the serum alpha fetoprotein (AFP) levels by genotyping both genes for Kindlin‐1 (three SNPs) and Kindlin‐2 (three SNPs) in Chinese hepatoma patients (*n* = 151). (C) Cytosine‐guanine dinucleotide (CpG) numbers in Kindlin‐1 gene in promotor and coding region— transcriptional start site (TSS) ± 2500 bp. (D) CpG numbers in Kindlin‐2 gene in promotor and coding region—TSS ± 2000 bp. (E) DNA methylation level of promoter region in Kindlin‐2 gene in Chinese healthy subjects (*n* = 8) and hepatoma patients (*n* = 8, including four male and four female patients).

Methylation of cytosine‐guanine dinucleotide (CpG) site is one of the epigenetic modification mechanisms involved in gene expression and transcriptional regulation in carcinogenesis.^8^ The CpG sites of Kindlin‐1 and Kindlin‐2 were further observed from the transcriptional start site (TSS) ± 2500 bp range. As shown in Figure 2C (Table S6), the number of CpG loci was densely distributed within the range of Kindlin‐1 gene from TSS ± 540 bp, which were 6 (−540 bp) and 31 (+540 bp), respectively. As shown in Figure 2D (Table S7), the number of CpG sites was densely distributed within the range of Kindlin‐2 gene from TSS ± 540 bp. These results suggest that CpG sites densely present near TSS of these two genes may provide the basis for DNA chemical modification to regulate gene expression.

We further compared methylation levels at CpG sites upstream of TSS between healthy subjects and hepatoma patients. As shown in Figure 2E (Table S8), there was no significant difference in the methylation level of CpG site in the promoter region of Kindlin‐2 gene between healthy subjects (*n* = 8) and hepatoma patients (*n* = 8, including four male and four female patients). The results suggest that the methylation degree of Kindlin‐2 gene in promoter region may not be involved in the occurrence and progression of hepatocarcinogenesis.

Whereafter, DNA methylation detection was extended to the whole genome to compare the healthy subjects and liver cancer patients after genotyping.^9^ Among the three functional SNPs of Kindlin‐1 gene, including Exon 7 (C>T, rs202037230) (Figure 3A), Exon 11 (C>T, rs2232074) (Figure 3B) and Exon 3 (A>G, rs16991866) (Figure 3C), DNA methylation levels of wild‐type homozygous genotype in hepatoma patients were significantly higher than those in healthy subjects (*p* < .01). However, no significant effect of Kindlin‐1 genotype on DNA methylation was observed among hepatoma patients (Table S9). Similarly, although there are no significant differences among patients, DNA methylation levels of Kindlin‐2 gene, including Exon 11 (C>T, rs777658527) (Figure 3D), Exon 7 (G>A, rs2357947) (Figure 3E) and Exon 4 (T>A/T>C/T>G, rs62003529) (Figure 3F) in hepatoma patients were significantly higher than those in healthy subjects (*p* < .01) (Table S10). Furthermore, the present results showed that DNA methylation levels were higher in men (*n* = 116) than in women (*n* = 15, *p* < .05), consistent with a higher number of male hepatoma patients (total *n* = 131). These results suggest that epigenetic modifications caused by DNA methylation of Kindlins are associated with hepatocarcinogenesis.

**FIGURE 3:**
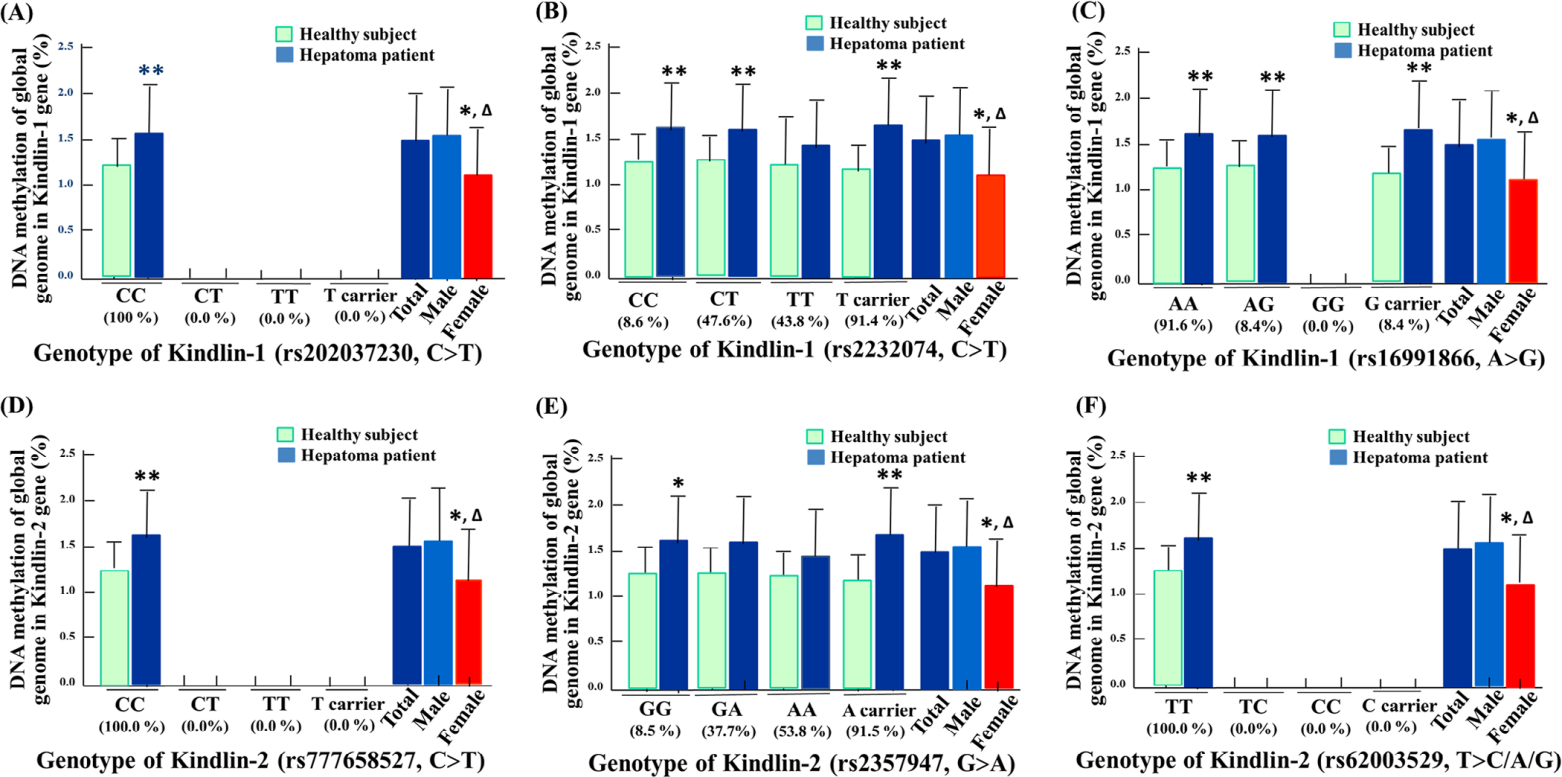
DNA methylation levels of global genome by genotyping for Kindlin‐1 and Kindlin‐2 genes between the healthy subjects and hepatoma patients. (A–C) DNA methylation level of global genome by genotyping for Kindlin‐1 gene in three single nucleotide polymorphisms (SNPs), including (A) Exon 7 (C>T, rs202037230), (B) Exon 11 (C>T, rs2232074) and (C) Exon 3 (A>G, rs16991866) as the wild‐type homozygotes, variant heterozygotes, variant homozygotes and variant allelic carriers (the sum of the variant heterozygote and variant homozygote genotypes), in Chinese healthy subjects (*n* = 116) and hepatoma patients (*n* = 151, *p* < .05 or <.01 compared with Chinese healthy group). (D–F) DNA methylation level of global genome by genotyping for Kindlin‐2 gene in three SNPs, including (D) Exon 11 (C>T, rs777658527), (E) Exon 7 (G>A, rs2357947) and (F) Exon 4 (T>A/T>C/T>G, rs62003529) as the wild‐type homozygotes, variant heterozygotes, variant homozygotes and variant allelic carriers (the sum of the variant heterozygote and variant homozygote genotypes), in Chinese healthy subjects (*n* = 116) and hepatoma patients (*n* = 151, *p* < .05 or <.01 compared with Chinese healthy group), and in male (*n* = 133) and female (*n* = 18, *p* < .05, compared with Chinese male group) in Chinese hepatoma patients. All data are shown as mean ± standard deviation (SD). ^*^ *p* < .05, ^**^ *p* < .01, compared with healthy subject group; Δ*p* < .05, compared with male group; NS: no significance.

However, whether epigenetic modification, especially DNA methylation, affects Kindlins gene expression and its causal relationship need to be further investigated. Furthermore, whether epigenetic modification of Kindlins affect the clinical outcome of hepatoma patients including tumour node metastasis staging or overall survival, its need to be confirmed by more exact method such as immunohistochemistry in future studies.^10^

In conclusion, the present results showed that epigenetic modification of Kindlins rather than genetic factors may be involved in hepatocarcinogenesis in the same genetic background Chinese population. Therefore, regulating the DNA methylation level of Kindlins may be a potential target for anti‐liver cancer drug therapy.

## AUTHOR CONTRIBUTIONS

Guoliang Zhang and Hongquan Zhang conceived and designed the experiments. Yang Yuan, Xiaona Dong, Shengju Yin, Haiyan Zhou, Guohui Li, Yan Tang and Xiaofan Wei performed the experiments and analyzed the data. Shengju Yin, Guoliang. Zhang, Xiaofan Wei and Hongquan Zhang wrote the paper. All authors approved the final version of the paper.

## CONFLICT OF INTEREST STATEMENT

The authors declare they have no conflicts of interest.

## ETHICS STATEMENT

Ethical informed consent exemptions were obtained from the Peking University Health Science Review Board (Permit Number: IRB00001052‐05101) and the Human Genetic Resources Management Office, Ministry of Science and Technology, China (Permit Number: [2018] 23‐189‐2559, https://fuwu.most.gov.cn/html/rlycjgcx/20181207/3031.html).

## Supporting information

Supporting Information

## Data Availability

All data generated or analysed during this study are included either in this article or in the supporting information files.
