# Supplementary material for: Focal adhesions Kindlin‐1 and Kindlin‐2 affected by epigenetic modifying in hepatocarcinogenesis
Source: Clin Transl Med. 2024 Jan 10;14(1):e1517. doi: 10.1002/ctm2.1517 (PMC10782135; doi:10.1002/ctm2.1517)
Supplement: Supplementary file 1 — Supporting Information [file CTM2-14-e1517-s001.doc]

**Focal adhesions Kindlin-1 and Kindlin-2 affected by epigenetic modifying in hepatocarcinogenesis**

Yang Yuan^1#^, Xiaona Dong^2#^, Shengju Yin^2^*, Guoliang Zhang^2^*, Haiyan Zhou^3^, Guohui Li^3^, Yan Tang^1^, Xiaofan Wei^1^*, and Hongquan Zhang^1^*

**Supporting Information**

**Supplemental Materials and Methods**

A full explanation of all methods used in this study can be found within the Supplemental Information.

**Study design:** The objective of this study was to investigate whether genetic and epigenetic variants of focal adhesion Kindlins (Kindlin-1 and Kindlin-2) influence hepatocarcinogenesis in healthy subjects and hepatocellular carcinoma patients in Chinese population. The subjects were divided into two groups including healthy subjects (n=116 cases) and hepatocellular carcinoma patients (n=241 cases). Next, functional single nucleotide polymorphisms (SNPs) in six variant sites in two genes were determined by the mass-spectrography method, including Exon 7 (C>T, rs202037230), Exon 11 (C>T, rs2232074) and Exon 3 (A>G, rs16991866) in Kindlin-1 gene, and Exon 11 (C>T, rs777658527), Exon 7 (G>A, rs2357947) and Exon 4 (T>A/T>C/T>G, rs62003529) in Kindlin-2 gene, respectively. Subsequently, DNA methylation status in cytosine-guanine dinucleotide (CpG) sites of the promoter region and genomic-wide were detected. Then, serumu alpha fetoprotein (AFP) levels by genotyping for Kindlin-1 and Kindlin-2 genes were evaluated in hepatoma patients. Finally, the potential causal relationship was investigated among genotypes, epigenotypes, and hepatocarcinogenesis phenotypes in Chinese subjects. This study provides a new explanation for the mechanism of epigenetics in hepatocarcinogenesis, and provides a new insight for potential drug treatment target of hepatocellular carcinoma.

**Ethics**

This study protocol was conducted in accordance with the Declaration of Helsinki and approved by the Ethics Committees at the Peking University Institutional Review Board (Permit Number: IRB00001052-05101), and Human Genetic Resources Management Office, Ministry of Science and Technology, China (Permit Number: [2018] 23-189-2559, https://fuwu.most.gov.cn/html/rlycjgcx/20181207/3031.html). All the participants provided written consent, which has been the review procedure of the ethics committee.

**Participants**

Total three hundreds and fifty-seven (357) Chinese Han ethnic subjects were recruited for the present research, in which there were one hundred and sixteen (116) healthy subjects and two hundred and forty-one (241) hepatocarcinoma patients, respectively. The clinical characteristics of healthy volunteers and hepatocellular carcinoma patients were showed in Supplementary Table S3.

**DNA extraction and genotyping**

Genomic DNA was extracted from EDTA-treated leukocytes in whole blood using QIAamp DNA Blood Mini Kits (Qiagen Inc., Valencia, California, USA). Single nucleotide polymorphisms (SNPs) in Kindlin-1 gene including Exon 7 (C>T, rs202037230), Exon 11 (C>T, rs2232074) and Exon 3 (A>G, rs16991866), and in Kindlin-2 gene including Exon 11 (C>T, rs777658527), Exon 7 (G>A, rs2357947) and Exon 4 (T>A/T>C/T>G, rs62003529) were determined in 357 Chinese individuals. All of 357 DNA samples were met the requirements for genotyping: DNA concentration was determined by NanoDrop 1000 (Waltham, U.S.A.) and was no less than 50 ng/μL. DNA total content was no less than 1 μg and the ranges of optical density (OD) 260/280 ratio were between 1.6 and 2.1 in all samples.

SNPs genotyping were performed by mass spectrometer method on Sequenom MassARRAY platform (Sequenom, San Diego, CA, USA) at CapitalBio Corporation (Beijing, China) according to the manufacturer’s protocol. Specific assays including a locus-specific PCR reaction based on a locus-specific primer extension reaction were designed using the MassARRAY Assay Design software package (v3.1) according to the manufacturer’s protocol. The detailed information regarding PCR primers, conditions, and products of PCR amplification, is listed in Supporting Information Table S11.

**Promoter region CpG sites methylation analysis**

Promoter region CpG sites methylation status of nine xenobiotic metabolic genes including Kindlin-2 gene were estimated via the Sequenom MassARRAY platform (CapitalBio, Beijing, China) according to the manufacturer’s protocol. The procedure of quantitative DNA methylation analysis of Kindlin-2 gene in promoter regions included bisulfite treatment of DNA, PCR amplification, *in vitro* transcription, RNA base-specific cleavage, and matrix-assisted laser desorption ionizationtime of flight mass spectrometry (MALDI-TOF-MS) analysis (MassARRAY Analyzer 4 system, Sequenom, San Diego, CA, USA) as described in previous publications [1]. The detailed information regarding primers, conditions and products of PCR amplification, and cytosine residue rates of bisulfite conversion in promoter region, is listed in Supporting InformationTable S12.

**Global genomic DNA methylation analysis**

Global genomic DNA methylation was detected with the Methylamp Global DNA Methylation Quantification Ultra Kit (Epigentek, New York, NY) according to manufacturer’s instructions. The methylated fractions of DNA are recognized by an anti-5-methylcytosine antibody and quantified by an enzyme-linked immunosorbent assay-like reaction as described in previous publications [1].

**Statistical analysis**

The frequencies of each polymorphism were assessed for deviation from Hardy–Weinberg equilibrium and were compared between different groups using Fisher’s exact test. Results are presented as mean ± standard deviation (SD) and analyzed by SPSS software version 18.0 (IBM SPSS, Armonk, New York, USA). Statistical significance of mean values was accessed by one-way analysis of variance (ANOVA). Independent *t*-test (2-tailed) was performed to evaluate the difference between two groups. *P* values less than 0.05 were considered statistically significant.

**REFERENCE**

1. L. Zhang, X. J. Miao, X. Wang, H. H. Pan, P. Li, H. Ren, Y. R. Jia, C. Lu, H. B. Wang, L. Yuan, G. L. Zhang, Antiproliferation of berberine is mediated by epigenetic modification of constitutive androstane receptor (CAR) metabolic pathway in hepatoma cells. *Sci. Rep.* 2016, *6,* 28116.

**Supporting Information**

**Supplementary data**

1. Table S1 Allelic frequencies of focal adhesion Kindlin-1 gene in single nucleotide polymorphism (SNP) including Exon 7 (C>T, rs202037230), Exon 11 (C>T, rs2232074) and Exon 3 (A>G, rs16991866) in Chinese healthy subjects and hepatoma patients compared with previous reported Caucasian and African populations.
2. Table S2 Allelic frequencies of focal adhesion Kindlin-2 gene in single nucleotide polymorphism (SNP) including Exon 11 (C>T, rs777658527), Exon 7 (G>A, rs2357947) and Exon 4 (T>A / T>C / T>G, rs62003529) in Chinese healthy subjects and hepatoma patients.
3. Table S3 Clinical characteristics of Chinese healthy subjects (n=116) and hepatocellular carcinoma patients (n=241).
4. Table S4 Serumu alpha fetoprotein (AFP) levels by genotyping for Kindlin-1 (3 SNPs) in hepatoma patients (n=161).
5. Table S5 Serumu alpha fetoprotein (AFP) levels by genotyping for Kindlin-2 (3 SNPs) in hepatoma patients (n=161).
6. Table S6 CpG numbers in promotor-coding region- Kindlin-1 –TSS ± 2000 bp.
7. Table S7 CpG numbers in promotor-coding region-Kindlin-2–TSS ± 2000 bp.
8. Table S8 DNA methylation level of promoter region by genotyping for Kindlin-2 (3 SNPs) in Chinese healthy subjects and hepatoma patients.
9. Table S9 DNA methylation level of global genome by genotyping for Kindlin-1 (3 SNPs) in Chinese healthy subjects and hepatoma patients.
10. Table S10 DNA methylation level of global genome by genotyping for Kindlin-2 (3 SNPs) in Chinese healthy subjects and hepatoma patients.
11. Table S11 -PCR primers for SNP genotyping in this study.
12. Table S12 - PCR primers for promotor CpG methylation in this study.
13. Figure S1-S2-CpG position in promotor DNA sequence in this study.

**Appendix TableS1-1 Table S1 -1**

**Table S1-1** Allelic frequencies of integrin tail binding protein (focal adhesion) Kindlin-1 gene Exon 7 (CT, rs202037230) single nucleotide polymorphism (SNP) in Chinese (healthy subjects and hepatoma patients) compared with previous reported Caucasian and African populations [1, 2].

|  | SNP |  | Frequencies % (n) | | | | | | | | | | | | |
| --- | --- | --- | --- | --- | --- | --- | --- | --- | --- | --- | --- | --- | --- | --- | --- |
| 11  （3-1） | Kindlin-1  rs202037230 |  | Chinese (This study) | |  |  |  | Caucasian | | |  |  | African | | |
|  |  |  | Healthy | Hepatoma |  |  | |  | |  |  | |  | |  |
|  | Exon 7, C>T | (n= 116) | | (n=241) | *P* value | | (n= 186048) | | ~~-~~ | *P* value | | (n=4954) | | (n=176) | *P* value |
|  | Allele |  | |  |  | |  | | |  | |  | | |  |
|  | C | 100.0 % (232/232) | | 100.0 % (482/482) | - | | 100.0 | | - | - | | 100.0 | | 100.0 | - |
|  | T | 0.0 % (0) | | 0.0 % (0) | - | | 0.0 | | - | - | | 0.0 | | 0.0 | - |

SNP: single nucleotide polymorphism.

[1] <https://www.ncbi.nlm.nih.gov/> SNP Database/ (2021)

[2] AlleleFrequencyAggregator (ALFA) Project (2020, NCBI)

**Table S1-2** Allelic frequencies of integrin tail binding protein (focal adhesion) Kindlin-1 gene Exon 11 (CT, rs2232074) single nucleotide polymorphism (SNP) in Chinese (healthy subjects and hepatoma patients) compared with previous reported Caucasian and African populations [1, 2].

|  | SNP | |  | Frequencies % (n) | | | | | | | | | | | | | | |
| --- | --- | --- | --- | --- | --- | --- | --- | --- | --- | --- | --- | --- | --- | --- | --- | --- | --- | --- |
| 12  (3-2) | Kindlin-1  rs2232074 | |  | Chinese (This study) | | | |  | Caucasian | | | | |  | African | | | |
|  |  | |  | Healthy | | Hepatoma |  |  |  | | |  | |  |  | | |  |
|  | Exon 11, C>T | | (n= 115) | | | (n=238) | *P* value | | (n=180444) | | (n=194) | | *P* value | | (n= 5166) | | (n= 186) | *P* value |
|  | Allele | |  | | |  |  | |  | | |  | | |  | | |  |
|  | C | 22.2 % (51/230) | | | 29.0 % (138/476) | | 0.141108 | | 62.2 | 67.7** | | *P*=0.000000 | | | 53.4 | 48.4**,^ΔΔ^ | | *P*=0.000000 |
|  |  |  |  |  |  |  |  |  |  |  |  |  |  |  |  |  |  | *P*=0.005244 |
|  | T | 77.8 % (179/230) | | | 71.0 % (338/476) | | 0.45398 | | 37.8 | 32.3** | | *P*=0.000000 | | | 46.6 | 51.6**, ^ΔΔ^ | | *P*=0.002055 |
|  |  |  |  |  |  |  |  |  |  |  |  |  |  |  |  |  |  | *P*=0.000502 |

SNP: single nucleotide polymorphism.

^*^*P* < 0.05, ^**^*P* < 0.01, compared with Chinese healthy subject group. ^Δ^*P* < 0.05, ^ΔΔ^*P* < 0.01, compared with Caucasian group.

[1] <https://www.ncbi.nlm.nih.gov/> SNP Database/ (2021)

[2] AlleleFrequencyAggregator (ALFA) Project (2020, NCBI).

**Appendix Table S1-3** Allelic frequencies of integrin tail binding protein (focal adhesion) Kindlin-1 (Fermt1) gene Exon 3 (AG, rs16991866) single nucleotide polymorphism (SNP) in Chinese (healthy subjects and hepatoma patients) compared with previous reported Caucasian and African populations [1, 2].

|  | SNP |  | Frequencies % (n) | | | | | | | | | | |
| --- | --- | --- | --- | --- | --- | --- | --- | --- | --- | --- | --- | --- | --- |
| 13  (3-3) | Kindlin-1  rs16991866 |  | Chinese (This study) | | |  | Caucasian | | |  | African | | |
|  |  |  | Healthy | Hepatoma |  |  |  | |  |  |  | |  |
|  | Exon 3, A>G | (n= 116) | | (n=241) | *P* value | (n= 190346) | | (n= 60) | *P* value | (n= 8968) | | (n= 302) | *P* value |
|  | Allele |  | |  |  |  | | |  |  | | |  |
|  | A | 94.4 % (219/232) | | 95.2 % (459/482) | 0.938980 | 90.6 | | 90.0 | - | 87.7 | | 86.4 | - |
|  | G | 5.6 % (13/232) | | 4.8 % (23/482) | 0.651534 | 9.4 | | 10.0 | - | 12.3 | | 13.6 | - |

SNP: single nucleotide polymorphism.

[1] <https://www.ncbi.nlm.nih.gov/> SNP Database/

[2] AlleleFrequencyAggregator (ALFA) Project (2020, NCBI).

**Appendix Table S2 Table S2**

**Table S2-1** Allelic frequencies of integrin tail binding protein (focal adhesion) Kindlin-2 gene Exon 11 (CT, rs777658527) single nucleotide polymorphism (SNP) in Chinese healthy subjects and hepatoma patients compared with previous reported Caucasian and African populations [1, 2].

|  | SNP |  | Frequencies % (n) | | | | | | | | | | | |
| --- | --- | --- | --- | --- | --- | --- | --- | --- | --- | --- | --- | --- | --- | --- |
| 14  (4-1) | Kindlin-2  rs777658527 |  | Chinese (This study) | | |  | Caucasian | | | |  | African | | |
|  |  |  | Healthy | Hepatoma |  |  |  | | |  |  |  | |  |
|  | Exon 11, C>T | (n= 116) | | (n=238) | *P* value | | (n= 9690) | | - | *P* value | | (n=2898) | (n=114) | *P* value |
|  | Alleles |  | |  |  | |  | | |  | |  |  |  |
|  | C | 100.0 % (232/232) | | 100.0 % (476/476) | - | | 100.0 | - | | - | | 100.0 | 100.0 | - |
|  | T | 0.0 % (0) | | 0.0 % (0) | - | | 0.0 | - | | - | | 0.0 | 0.0 | - |

SNP: single nucleotide polymorphism.

[1] <https://www.ncbi.nlm.nih.gov/> SNP Database (2021)

[2] AlleleFrequencyAggregator (ALFA) Project (2020, NCBI)

**Appendix Table S2-2** Allelic frequencies of integrin tail binding protein (focal adhesion) Kindlin-2 gene Exon 7 (GA, rs2357947) single nucleotide polymorphism (SNP) in Chinese healthy subjects and hepatoma patients compared with previous reported Caucasian and African populations [1, 2].

|  | SNP | |  | Frequencies % (n) | | | | | | | | | | | | |
| --- | --- | --- | --- | --- | --- | --- | --- | --- | --- | --- | --- | --- | --- | --- | --- | --- |
| 15  (4-2) | Kindlin-2  rs2357947 | |  | Chinese (This study) | | | |  | | Caucasian | | |  | African | | |
|  |  | |  | Healthy | | Hepatoma |  |  | |  | |  |  |  | |  |
|  | Exon 7, G>A | | (n= 116) | | | (n=237) | *P* value | (n= 152346) | | | (n= 63) | *P* value | | (n= 7968) |  | *P* value |
|  | Alleles | |  | | |  |  |  | | |  |  | |  |  |  |
|  | G | 28.5 % (66/232) | | | 30.0 % (142/474) | | 0.759858 | | 9.95 | | 10.5** | *P*=0.001208 | | 48.8 | 55.3**, ^ΔΔ^ | *P*=0.000020 |
|  |  |  |  |  |  |  |  |  |  |  |  |  |  |  |  | *P*=0.000020 |
|  | A | 71.5 % (166/232) | | | 70.0 % (332/474) | | 0.862831 | | 90.05 | | 89.5* | *P*=0.006924 | | 51.2 | 44.7**, ^ΔΔ^ | *P*=0.000233 |
|  |  |  |  |  |  |  |  |  |  |  |  |  |  |  |  | *P*=0.000000 |

SNP: single nucleotide polymorphism.

^*^*P* < 0.05, ^**^*P* < 0.01, compared with Chinese healthy subject group. ^Δ^*P* < 0.05, ^ΔΔ^*P* < 0.01, compared with Caucasian group.

[1] <https://www.ncbi.nlm.nih.gov/> SNP Database/ (2021)

[2] AlleleFrequencyAggregator (ALFA) Project (2020, NCBI)

**Appendix Table S2-3** Allelic frequencies of integrin tail binding protein (focal adhesion) Kindlin-2 gene Exon 4 (T>A, rs62003529) single nucleotide polymorphism (SNP) in Chinese healthy subjects and hepatoma patients compared with previous reported Caucasian and African populations [1, 2].

|  | SNP |  | Frequencies % (n) | | | | | | | | | | | | | | | | | |
| --- | --- | --- | --- | --- | --- | --- | --- | --- | --- | --- | --- | --- | --- | --- | --- | --- | --- | --- | --- | --- |
| 16  (4-3) | Kindlin-2  rs62003529 |  | Chinese (This study) | | | |  | Caucasian | | | | |  | African | | | | | |  |
|  |  |  | Healthy | Hepatoma | | |  |  | | |  | |  |  | | |  | | |  |
|  | Exon 4, T >A/C/G | | (n= 116) | (n=241) | | *P* value | | (n=36510) | | (n=126) | | *P* value | | (n=6174) | | (n=206) | | | *P* value | |
|  | Alleles |  | |  | |  |  | |  | |  | | |  | |  | |  | | |
|  | T | 100.0 % (232/232) | | | 100.0 % (482/482) | | - | 80.1 | 79.0 | | *P*=0.075045 | | | 91.2 | 97.6 | | | - | | |
|  | A/C/G | 0.0 % (0) | | 0.0 % (0) | | | - | 19.9 | 21.0** | | *P*=0.000000 | | | 8.8 | 2.4 ^ΔΔ^ | | | *P*=0.000000 | | |

SNP: single nucleotide polymorphism.

^*^*P* < 0.05, ^**^*P* < 0.01, compared with Chinese healthy subject group. ^Δ^*P* < 0.05, ^ΔΔ^*P* < 0.01, compared with Caucasian group.

[1] <https://www.ncbi.nlm.nih.gov/> SNP Database/

[2] AlleleFrequencyAggregator (ALFA) Project (2020, NCBI)

**Supplementary data Table S3**

**~~Appendix Table S3-~~** ~~Clinical characteristics of Chinese healthy subjects (n = 116) and hepatoma patients (n = 241).~~

|  | | | Characteristics | | | | | |
| --- | --- | --- | --- | --- | --- | --- | --- | --- |
|  |  | |  | Obs (n) | % | Max | Min | Mean±SD |
| 1 | Healthy subjects (n=116) | | | |  |  |  |  |
|  | | Gender | |  |  |  |  |  |
|  | | Female | | 64 | 55.2 |  |  |  |
|  | | Male | | 52 | 44.8 |  |  |  |
|  |  | Age | | 116 |  | 83 | 34 | 54.47±11.01 |
| 2 | Hepatoma patients (n = 241) | | | |  |  |  |  |
|  | | Gender | |  |  |  |  |  |
|  | | Female | | 25 | 10.4 |  |  |  |
|  | | Male | | 216 | 89.6 |  |  |  |
|  | | Age | | 241 |  | 79 | 33 | 55.98±9.61 |
|  | | AFP (ng/mL) | | 229 |  | 60500.0 | 0.89 | 2121±7127.35 |
|  | | ≤ 400 ng/mL | | 151 | 65.9 | 400 | 0.89 | 46.67±90.78 |
|  | | > 400 ng/mL | | 78 | 34.1 | 60500.0 | 401 | 24074.93± 26449.19 |
|  | | HBV (copies/mL) | | 219 |  | 82300000 | 0 | 1469067± 8485344 |
|  | | ≤5×10^2^ copies/mL | | 130 | 59.4 | 500 | 0 | 294.27 ± 243.27 |
|  | | >5×10^2^ copies/mL | | 89 | 40.6 | 82300000 | 501 | 3614464 ± 13058496 |

Obs: observed; %, percentage; Max, maximum; Min, minimum.

AFP: alpha fetoprotein

HBV: Hepatitis B virus

**Appendix Table S4- Table S4**

**~~Table S4-1~~** ~~Serumu alpha fetal protein (AFP) levels by genotyping of focal adhesion Kindlin-1 in Exon 7 (CT, rs202037230) in hepatoma patients (151).~~

|  | SNP |  | Frequencies |  | Alpha fetal protein (AFP) | |
| --- | --- | --- | --- | --- | --- | --- |
| 11  (3-1) | Kindlin-1 (Fermt1)  rs202037230 |  | % (n) |  | ng/mL | *P* value |
|  | (Exon 7, C>T) |  |  | |  |  |
|  | Genotype |  |  | |  |  |
|  | wt/wt (CC) |  | 100.0 % (151/151) | | 8427.066±19511.11 |  |
|  | wt/mu (CT) |  | 0.0 % (0/151) | | 0.0±0.0 |  |
|  | mu/mu (TT) |  | 0.0 % (0/151) | | 0.0±0.0 |  |
|  | mu carrier (CT+TT) |  | 0.0 % (0/151) | | 0.0±0.0 |  |
|  | Gender |  |  | |  |  |
|  | Total |  | 151 | | 8427.066±19511.11 |  |
|  | Male |  | 88.1 % (133) | | 8191.854±19053.1 | 0.9184 |
|  | Female |  | 11.9 % (18) | | 10165.02±23164.48 | 0.7268 |

SNP, single nucleotide polymorphism;

wt/wt (CC, Wild-type homozygous; wt/mu (CT), Variant heterozygous; mu/mu (TT), Variant homozygous; mu carrier (CT+TT), Variant allelic carrier.

focal adhesion Kindlin-1

AFP: alpha fetoprotein

**Table S4-2** Serumu alpha fetal protein (AFP) levels by genotyping of focal adhesion Kindlin-1 in Exon 11 (CT, rs2232074) in hepatoma patients (149).

|  | SNP |  | Frequencies |  | Alpha fetal protein (AFP) | |
| --- | --- | --- | --- | --- | --- | --- |
| 12  (3-2) | Kindlin-1 (Fermt1)  rs2232074 |  | % (n) |  | ng/mL | *P* value |
|  | (Exon 11, C>T) |  |  | |  |  |
|  | Genotype |  |  | |  |  |
|  | wt/wt (CC) |  | 7.4 % (11/149) | | 11173.35±24389.98 |  |
|  | wt/mu (CT) |  | 50.3 % (75/149) | | 7238.298±17719.56 | 0.5150 |
|  | mu/mu (TT) |  | 42.3 % (63/149) | | 9553.36±21089.79 | 0.8189 |
|  | mu carrier (CT+TT) |  | 92.6 % (138/149) | | 8110.401±19180.24 | 0.6183 |
|  | Gender |  |  | |  |  |
|  | Total |  | 149 | | 8507.657 ± 19629.62 |  |
|  | Male |  | 87.9 % (131) | | 8279.928 ± 19185.2 | 0.9221 |
|  | Female |  | 12.1 % (18) | | 10165.02 ± 23164.48 | 0.7405 |

SNP: single nucleotide polymorphism;

wt/wt (CC): Wild-type homozygous; wt/mu (CT): Variant heterozygous; mu/mu (TT): Variant homozygous; mu carrier (CT+TT): Variant allelic carrier.

focal adhesion Kindlin-1

AFP: alpha fetoprotein

**Table S4-3** Serumu alpha fetal protein (AFP) levels by genotyping of focal adhesion Kindlin-1 in Exon 3 (AG, rs16991866) in hepatoma patients (151).

|  | SNP |  | Frequencies |  | Alpha fetal protein (AFP) | |
| --- | --- | --- | --- | --- | --- | --- |
| 13  (3-3) | Kindlin-1 (Fermt1)  rs16991866 |  | % (n) |  | ng/mL | *P* value |
|  | (Exon 3, A>G) |  |  | |  |  |
|  | Genotype |  |  | |  |  |
|  | wt/wt (AA) |  | 90.0 % (136/151) | | 8349.896±19415.14 |  |
|  | wt/mu (AG) |  | 10.0 % (15/151) | | 9126.741±21055.09 | 0.8842 |
|  | mu/mu (GG) |  | 0.0 % (0/151) | | 0.0±0.0 |  |
|  | mu carrier (AG+GG) |  | 10.0 % (15/151) | | 9126.741±21055.09 | 0.8842 |
|  | Gender |  |  | |  |  |
|  | Total |  | 151 | | 8427.066 ±19511.11 |  |
|  | Male |  | 88.1 % (133) | | 8098.318±19074.35 | 0.8863 |
|  | Female |  | 11.9 % (18) | | 10165.02±23164.48 | 0.7268 |

SNP, single nucleotide polymorphism;

wt/wt (AA), Wild-type homozygous; wt/mu (AT), Variant heterozygous; mu/mu (TT), Variant homozygous; mu carrier (AT+TT), Variant allelic carrier.

focal adhesion Kindlin-1

AFP: alpha fetoprotein

**Appendix Table S5 Table S5**

**Table S5-1** Serumu alpha fetal protein (AFP) levels by genotyping of focal adhesion Kindlin-2 in Exon 11 (CT, rs777658527) in Chinese hepatoma patients (n=148).

|  | SNP |  | Frequencies |  | Alpha fetal protein (AFP) | |
| --- | --- | --- | --- | --- | --- | --- |
| 14  (4-1) | Kindlin-2 (Fermt2)  rs777658527 |  | % (n) |  | ng/mL | *P* value |
|  | (Exon 11, C>T) |  |  | |  |  |
|  | Genotype |  |  | |  |  |
|  | wt/wt (CC) |  | 100.0 % (148/148) | | 8533.736±19684.24 |  |
|  | wt/mu (CT) |  | 0.0 % (0/148) | | 0.0±0.0 |  |
|  | mu/mu (TT) |  | 0.0 % (0/148) | | 0.0±0.0 |  |
|  | mu carrier (CT+TT) |  | 0.0 % (0/148) | | 0.0±0.0 |  |
|  | Gender |  |  | |  |  |
|  | Total |  | 148 | | 8533.736±19684.24 |  |
|  | Male |  | 87.8 % (130) | | 8307.867±19245.73 | 0.9232 |
|  | Female |  | 12.2 % (18) | | 10165.02±23164.48 | 0.7452 |

SNP, single nucleotide polymorphism;

wt/wt (CC), Wild-type homozygous; wt/mu (CT), Variant heterozygous; mu/mu (TT), Variant homozygous; mu carrier (CT+TT), Variant allelic carrier.

focal adhesion Kindlin-2

AFP: alpha fetoprotein.

**Table S5-2** Serumu alpha fetal protein (AFP) levels by genotyping of focal adhesion Kindlin-2 in Exon 7 (GA, rs2357947) in Chinese hepatoma patients (n=151).

|  | SNP |  | Frequencies |  | Alpha fetal protein (AFP) | |
| --- | --- | --- | --- | --- | --- | --- |
| 15  (4-2) | Kindlin-2 (Fermt2)  rs2357947 |  | % (n) |  | ng/mL | *P* value |
|  | (Exon 7, G>A) |  |  | |  |  |
|  | Genotype |  |  | |  |  |
|  | wt/wt (GG) |  | 8.6 % (13/151) | | 11909.23±23286.81 |  |
|  | wt/mu (GA) |  | 38.4 % (58/151) | | 9280.977±20813.52 | 0.6884 |
|  | mu/mu (AA) |  | 53 % (80/151) | | 7261.733±17990.91 | 0.4100 |
|  | mu carrier (GA+AA) |  | 91.4 % (138/151) | | 8110.401±19180.24 | 0.5039 |
|  | Gender |  |  | |  |  |
|  | Total |  | 151 | | 8427.066±19511.11 |  |
|  | Male |  | 88.1% (133) | | 8191.854±19053.1 | 0.9184 |
|  | Female |  | 11.9 % (18) | | 10165.02±23164.48 | 0.7109 |

SNP, single nucleotide polymorphism;

wt/wt (GG), Wild-type homozygous; wt/mu (GA), Variant heterozygous; mu/mu (AA), Variant homozygous; mu carrier (GA+AA), Variant allelic carrier.

focal adhesion Kindlin-2

AFP: alpha fetoprotein.

**Table S5-3** Serumu alpha fetal protein (AFP) levels by genotyping of focal adhesion Kindlin-2 in Exon 4 (T>A / T>C / T>G, rs62003529) in Chinese hepatoma patients (n=151).

|  | SNP |  | Frequencies |  | Alpha fetal protein (AFP) | |
| --- | --- | --- | --- | --- | --- | --- |
| 16  (4-3) | Kindlin-2 (Fermt2)  rs62003529 |  | % (n) |  | ng/mL | *P* value |
|  | (Exon 4, T>C) |  |  | |  |  |
|  | Genotype |  |  | |  |  |
|  | wt/wt (TT) |  | 100.0 % (151/151) | | 8427.066±19511.11 |  |
|  | wt/mu (TC) |  | 0.0 % (0/151) | | 0.0±0.0 |  |
|  | mu/mu (CC) |  | 0.0 % (0/151) | | 0.0±0.0 |  |
|  | mu carrier (TC +CC) |  | 0.0 % (0/151) | | 0.0±0.0 |  |
|  | Gender |  |  | |  |  |
|  | Total |  | 151 | | 8427.066±8427.066 |  |
|  | Male |  | 88.1 % (133) | | 8191.854±19053.1 | 0.9184 |
|  | Female |  | 11.9 % (18) | | 10165.02±23164.48 | 0.5227 |

SNP, single nucleotide polymorphism;

wt/wt (TT), Wild-type homozygous; wt/mu (TC), Variant heterozygous; mu/mu (CC), Variant homozygous; mu carrier (TC +CC), Variant allelic carrier.

focal adhesion Kindlin-2

AFP: alpha fetoprotein.

**Appendix Table S6 Table S6**

**Table S6** Numbers of cytosine-guanine dinucleotide (CpG) sites in promotor (TSS, -1~-2500 bp) and coding region (TSS, +1~ +2500 bp) of focal adhesion Kindlin-1 (Fermt1) gene in human.

|  | Gene | Location of CpG sites | Number of CpG sites | |
| --- | --- | --- | --- | --- |
| 3 | Kindlin-1 | (From transcription start site,  TSS, ±1 bp ~) | Promotor region | Coding region |
|  |  |  | Upstream  from TSS  (-1~-2500 bp) | Downstream  from TSS  (+1~+2500 bp) |
|  |  | ±1~±540 (481+59) =540 bp | 6 | 31 |
|  |  | ±541~±1020 (961 +59) =540 bp | 6 | 36 |
|  |  | ±1021~±1500 (1441+59) =540 bp | 6 | 9 |
|  |  | ±1501~±1980 (1921+59) =540 bp | 1 | 0 |
|  |  | ±1981~±2460 (2401+59) =540 bp | 7 | 6 |

Kindlin-1: focal adhesion (Fermt1).

TSS: transcription start site.

CpG: cytidine-phosphate-guanosine dinucleotides,

bp: base pair.

**Appendix Table S7 Table S7**

**Table S7** Numbers of cytosine-guanine dinucleotide (CpG) sites in promotor (TSS, -1~ -2500 bp) and coding region (TSS, +1~ +2500 bp) of focal adhesion Kindlin-2 (Fermt2) gene in human.

|  | Gene | Location of CpG sites | Number of CpG sites | | | |
| --- | --- | --- | --- | --- | --- | --- |
| 4 | Kindlin-2 | (From transcription start site,  TSS, ± 1 bp ~) | | Promotor region | | Coding region |
|  |  |  | Upstream  from TSS  (-1~ -2500 bp) | | Downstream  from TSS  (+1~ +2500 bp) | |
|  |  | ±1~±540 (481+59) =540 bp | 38 | | 71 | |
|  |  | ±541~±1020 (961 +59) =540 bp | 3 | | 16 | |
|  |  | ±1021~±1500 (1441+59) =540 bp | 1 | | 14 | |
|  |  | ±1501~±1980 (1921+59) =540 bp | 0 | | 14 | |
|  |  | ±1981~±2460 (2401+59) =540 bp | 1 | | 6 | |

Kindlin-2: focal adhesion (Fermt2).

TSS: transcription start site.

CpG: cytidine-phosphate-guanosine dinucleotides,

bp: base pair.

**Appendix Table S8 Table S8**

**Appendix Table S8** DNA methylation level of promoter region in Kindlin-2 gene in Chinese healthy subjects (n=8) and hepatoma patients (n=16).

|  | |  |  |  | Gender | Age | AFP  (ng/mL) |  |  | DNA Methylation Level (%)  Kindlin-2 Promotor Region (74 CpG sites, 23 CpG units) | | | | | | | |
| --- | --- | --- | --- | --- | --- | --- | --- | --- | --- | --- | --- | --- | --- | --- | --- | --- | --- |
|  |  |  |  |  |  |  |  |  | Group 0  (G0) | Group 1  (G1) | Group 2  (G2) | Group 3  (G3) | Group 4  (G4) | Group 5  (G5) | Group 6  (G6) | Group 7  (G7) | Group 8  (G8) |
| 1 | | 129 |  | H-45-F | F | 69 | <20 | 0.052609± 0.05233 | | G1-0 | G2-2 |  |  |  |  |  |  |
| 2 | | 177 |  | H-61-F | F | 46 | <20 | 0.037273± 0.02848 | | G1-0 | G2-2 |  |  |  |  |  |  |
| 3 | | 180 |  | H-62-F | F | 36 | <20 | 0.05913± 0.046896 | | G1-0 | G2-2 |  |  |  |  |  |  |
| 4 | | 350 |  | H-116-F | F | 54 | <20 | 0.04087± 0.043266 | | G1-0 | G2-2 |  |  |  |  |  |  |
|  | |  |  |  |  |  |  |  | |  | 0.04747±  0.010164  (n=4) |  |  |  |  |  |  |
| 5 | | 258 |  | H-88-M | M | 65 | <20 | 0.038261± 0.04097 | | G1-0 | G2-1 |  |  |  |  |  |  |
| 6 | | 264 |  | H-90-M | M | 57 | <20 | 0.056957± 0.05716 | | G1-0 | G2-1 |  |  |  |  |  |  |
| 7 | | 288 |  | H-98-M | M | 45 | <20 | 0.043478± 0.05348 | | G1-0 | G2-1 |  |  |  |  |  |  |
| 8 | | 297 |  | H-99 (113)-M | M | 61 | <20 | 0.042609± 0.07973 | | G1-0 | G2-1 |  |  |  |  |  |  |
|  | |  |  |  |  |  |  | 0.039623±  0.013826  (n=8) | | 0.04533±  0.00808  (n=4)  (*P*=0.75299) |  |  |  |  |  |  |  |
| 9 | | 251 |  | C-168-1-F | F | 71 | 8.59 | 0.02826± 0.035375 | | G1-1 |  | G3-2 | G4-1 | G5-2 |  |  | G8-1 |
| 10 | | 209 |  | C-140-1-F | F | 65 | 59.62 | 0.068636±  0.06144 | | G1-1 |  | G3-2 | G4-1 | G5-2 |  |  | G8-1 |
| 11 | | 301 |  | C-201-1-F | F | 45 | 3.45 | 0.01913± 0.032039 | | G1-1 |  | G3-2 | G4-1 | G5-2 |  |  | G8-1 |
| 12 | | 281 |  | C-188-1-F | F | 49 | 2.58 | 0.025652±  0.03501 | | G1-1 |  | G3-2 | G4-1 | G5-2 |  |  | G8-1 |
|  | |  |  |  |  |  |  |  | |  |  |  |  | 0.03542±  0.022475  (n=4-F) |  |  | 0.03837±  0.011787  (n=4) |
| 13 | | 235 |  | C-157-1-M | M | 39 | 182.8 | 0.042727± 0.04256 | | G1-1 |  | G3-1 | G4-1 | G5-1 |  | G7-1 |  |
| 14 | | 125 |  | C-84-1-M | M | 42 | 1.77 | 0.04652± 0.072777 | | G1-1 |  | G3-1 | G4-1 | G5-1 |  | G7-1 |  |
| 15 | | 155 |  | C-104-1-M | M | 60 | 239.9 | 0.05±  0.036556 | | G1-1 |  | G3-1 | G4-1 | G5-1 |  | G7-1 |  |
| 16 | | 149 |  | C-100-1-M | M | 42 | 3.06 | 0.054783± 0.05814 | | G1-1 |  | G3-1 | G4-1 | G5-1 |  | G7-1 |  |
|  | |  |  |  |  |  | 62.72125±  94.98541  (n=8) |  | |  |  |  | 0.037283±  0.010972  (n=8) | 0.048508±  0.00513  (n=4-M)  (*P*=0.3316) |  | 0.036196±  0.011781  (n=4) |  |
| 17 | | 25 |  | C-17-2-F | F | 45 | >60500.0 | 0.053478± 0.03996 | | G1-1 |  | G3-2 | G4-2 |  | G6-2 |  | G8-2 |
| 18 | | 92 |  | C-62-2-F | F | 49 | 38991 | 0.025217± 0.02826 | | G1-1 |  | G3-2 | G4-2 |  | G6-2 |  | G8-2 |
| 19 | | 151 |  | C-101-2-F | F | 38 | >60500.0 | 0.04±  0.033845 | | G1-1 |  | G3-2 | G4-2 |  | G6-2 |  | G8-2 |
| 20 | | 133 |  | C-89-2-F | F | 45 | >60500.0 | 0.034783± 0.03883 | | G1-1 |  | G3-2 | G4-2 |  | G6-2 |  | G8-2 |
|  | |  |  |  |  |  |  |  | |  |  | 0.036895±  0.016688  (n=8-F-2) |  |  | 0.03837±  0.011787  (n=4) |  | 0.03542±  0.022475  (n=4)  (*P*=0.82631) |
| 21 | | 148 |  | C-99-2-M | M | 59 | 1609 | 0.034783± 0.03872 | | G1-1 |  | G3-1 | G4-2 |  | G6-1 | G7-2 |  |
| 22 | | 169 |  | C-113-2-M | M | 57 | >60500.0 | 0.041739± 0.03473 | | G1-1 |  | G3-1 | G4-2 |  | G6-1 | G7-2 |  |
| 23 | | 172 |  | C-115-2-M | M | 50 | 3374 | 0.047826± 0.05485 | | G1-1 |  | G3-1 | G4-2 |  | G6-1 | G7-2 |  |
| 24 | | 215 |  | C-144-2-M | M | 61 | 509.1 | 0.020435± 0.04084 | | G1-1 |  | G3-1 | G4-2 |  | G6-1 | G7-2 |  |
|  |  | |  |  |  |  |  | 35810.39±  29072.47  (n=8)  17936.55±  27114.7  (n=16) | | 0.046398±  0.008578  (n=16)  (*P*=0.15570) |  | 0.042352±  0.010681  (n=8-F-2)  (*P*=0.45118) | 0.041964±  0.016634  (n=8)  (*P*=0.51883) |  | 0.036196±  0.011781  (n=4)  (*P*=0.80298) | 0.048508±  0.00513  (n=4)  (*P*=0.12610) |  |

F: Female; M: male; H: healthy; C: cancer;

AFP: alpha fetalprotein; CpG: cytimidine-guanine dinucleotide

**Appendix Table S9 Table S9**

**~~Appendix Table S9-1~~** ~~DNA methylation levels of global genome of focal adhesion Kindlin-1 (Fermt1) gene by genotyping for Exon 7 (CT, rs202037230) in Chinese healthy subjects (n=108) and hepatoma patients (n=131).~~

|  | SNP |  | Healthy subjects  (n= 108) | | | |  | Hepatoma patients  (n= 131) | | | | | |
| --- | --- | --- | --- | --- | --- | --- | --- | --- | --- | --- | --- | --- | --- |
| 11  (3-1) | Kindlin-1 (Fermt1)  rs202037230 |  | Frequencies | DNA metylation  of global genome | | *P* value | | | Frequencies | DNA metylation  of global genome | *P* value | *P* value | |
|  | (Exon 7, C>T) |  | % (n) | (%) |  | |  | % (n) | | (%) |  | |  |
|  | Genotype |  |  |  |  | |  |  | |  |  | |  |
|  | wt/wt (CC) | 100.0 % (108/108) | | 1.292±0.306 |  | |  | 100.0 % (131/131) | | 1.521±0.655 ^# # #^ | 0.0010 | |  |
|  | wt/mu (CT) |  | 0.0 % (0) | 0.0±0.0 |  | |  | 0.0 % (0) | | 0.0±0.0 |  | |  |
|  | mu/mu (TT) |  | 0.0 % (0) | 0.0±0.0 |  | |  | 0.0 % (0) | | 0.0±0.0 |  | |  |
|  | mu carrier (CT+TT) |  | 0.0 % (0) | 0.0±0.0 |  | |  | 0.0 % (0) | | 0.0±0.0 |  | |  |
|  | Allele |  |  |  |  | |  |  | |  |  | |  |
|  | C | 100.0 % (216/216) | |  |  | |  | 100.0 % (262/262) | |  |  | |  |
|  | T |  | 0.0 % (0) |  |  | |  | 0.0 % (0) | |  |  | |  |
|  | Gender |  |  |  |  | |  |  | |  |  | |  |
|  | Total |  | 108 | 1.292±0.306 |  | |  | 131 | | 1.521±0.655 ^# # #^ | 0.0010 | |  |
|  | Male |  | 39.8 % (43) | 1.306±0.312 |  | |  | 88.5 % (116) | | 1.573±0.645 ^#^ | 0.0102 | |  |
|  | Female |  | 60.2 % (65) | 1.283±0.303 |  | |  | 11.5 % (15) | | 1.118±0.609 ^Δ^ | 0.0108 | |  |

wt/wt (CC), Wild-type homozygous; wt/mu (CT), Variant heterozygous; mu/mu (TT), Variant homozygous; mu carrier (CT+TT), Variant allelic carrier.

SNP: single nucleotide polymorphism.

^*^*P* < 0.05, ^#^*P* < 0.05, ^# #^*P* < 0.01, compared with healthy subject groups. ^Δ^*P* < 0.05, compared with male group.

º*P* < 0.05, compared with wild-type CC homozygous genotypes.

**~~Appendix Table S9-2~~** ~~DNA methylation levels of global genome of focal adhesion Kindlin-1 (Fermt1) gene by genotyping for Exon 11 (CT, rs2232074) in Chinese healthy subjects (n=107) and hepatoma patients (n=128).~~

|  | SNPs |  | Healthy subjects  (n= 107) | | | |  | Hepatoma patients  (n= 128) | | |  |  |
| --- | --- | --- | --- | --- | --- | --- | --- | --- | --- | --- | --- | --- |
| 12  (3-2) | Kindlin-1 (Fermt1)  rs2232074 |  | Frequencies | DNA metylation  of global genome | | *P* value | | | Frequencies | DNA metylation  of global genome | *P* value | *P* value |
|  | Exon 11, C>T |  | % (n) | (%) |  | |  | % (n) | | (%) |  |  |
|  | Genotype |  |  |  |  | |  |  | |  |  |  |
|  | wt/wt (CC) |  | 0.9 % (1/107) | 1.90 |  | |  | 8.6 % (11/128) | | 1.561±0.766 | - |  |
|  | wt/mu (CT) |  | 40.2 % (43/107) | 1.292±0.294 |  | |  | 47.6 % (61/128) | | 1.616±0.616 ^# #^ | 0.0018 |  |
|  | mu/mu (TT) |  | 58.9 % (63/107) | 1.290±0.305 |  | |  | 43.8 % (56/128) | | 1.416±0.681 | 0.1871 |  |
|  | mu carrier (CT+TT) | 99.1 % (106/107) | | 1.291±0.299 |  | |  | 91.4 % (117/128) | | 1.52±0.652 ^# #^ | 0.0011 |  |
|  | Allele |  |  |  |  | |  |  | |  |  |  |
|  | C |  | 21.0 % (45/214) |  |  | |  | 32.4 % (83/256) | |  |  |  |
|  | T | 79.0 % (169/214) | |  |  | |  | 67.6 % (173/256) | |  |  |  |
|  | Gender |  |  |  |  | |  |  | |  |  |  |
|  | Total |  | 107 | 1.297±0.304 |  | |  | 128 | | 1.523±0.648^# #^ | 0.0011 |  |
|  | Male |  | 45.8 % (49) | 1.278±0.308 |  | |  | 88.3 % (113) | | 1.524±0.650 ^#^ | 0.0125 |  |
|  | Female |  | 54.2 % (58) | 1.312±0.301 |  | |  | 11.7 % (15) | | 1.118±0.609 ^Δ^ | 0.0238 |  |

wt/wt (CC), Wild-type homozygous; wt/mu (CT), Variant heterozygous; mu/mu (TT), Variant homozygous; mu carrier (CT+TT), Variant allelic carrier.

SNPs: single nucleotide polymorphisms.

^*^*P* < 0.05, ^#^*P* < 0.05, ^# #^*P* < 0.01, compared with healthy subject groups. ^Δ^*P* < 0.05, compared with male group.

º*P* < 0.05, compared with wild-type CC homozygous genotypes.

**~~Appendix Table S9-3~~** ~~DNA methylation levels of global genome of focal adhesion Kindlin-1 (Fermt1) gene by genotyping for Exon 3 (AG, rs16991866) in Chinese healthy subjects (n=108) and hepatoma patients (n=131).~~

|  | SNP |  | Healthy subjects  (n= 108) | |  | |  | Hepatoma patients  (n=131) | | |  |  |
| --- | --- | --- | --- | --- | --- | --- | --- | --- | --- | --- | --- | --- |
| 13  (3-3) | Kindlin-1 (Fermt1)  rs16991866 |  | Frequencies | DNA metylation  of global genome | | *P* value | | | Frequencies | DNA metylation  of global genome | *P* value | *P* value |
|  | Exon 3, A>G |  | % (n) | (%) |  | |  | % (n) | | (%) |  |  |
|  | Genotype |  |  |  |  | |  |  | |  |  |  |
|  | wt/wt (AA) |  | 88.0 % (95/108) | 1.284±0.302 |  | |  | 91.6 % (120/131) | | 1.506±0.659 ^# #^ | 0.0027 |  |
|  | wt/mu (AG) |  | 12.0 % (13/108) | 1.35±0.337 |  | |  | 8.4 % (11/131) | | 1.645±0.646 | 0.1652 |  |
|  | mu/mu (GG) |  | 0.0 % (0) | 0.0±0.0 |  | |  | 0.0 % (0) | | 0.0±0.0 |  |  |
|  | mu carrier (AG+GG) |  | 12.0 % (13/108) | 1.35±0.337 |  | |  | 8.4 % (11/131) | | 1.645±0.646 | 0.1652 |  |
|  | Allele |  |  |  |  | |  |  | |  |  |  |
|  | A |  | 94.0 % (203/216) |  |  | |  | 95.8 % (251/262) | |  |  |  |
|  | G |  | 6.0 % (13/216) |  |  | |  | 4.2 % (11/262) | |  |  |  |
|  | Gender |  |  |  |  | |  |  | |  |  |  |
|  | Total |  | 108 | 1.292±0.306 |  | |  | 131 | | 1.518±0.656 ^# #^ | 0.0011 |  |
|  | Male |  | 45.4 % (49) | 1.278±0.308 |  | |  | 88.55 % (116) | | 1.570±0.647 ^# #^ | 0.0030 |  |
|  | Female |  | 54.6 % (59) | 1.304±0.305 |  | |  | 11.45 % (15) | | 1.118±0.609 ^Δ^ | 0.0978 | 0.0116 |

wt/wt (AA), Wild-type homozygous; wt/mu (AG), Variant heterozygous; mu/mu (GG), Variant homozygous; mu carrier (AG+GG), Variant allelic carrier. SNP: single nucleotide polymorphism.

^*^*P* < 0.05, ^#^*P* < 0.05, ^# #^*P* < 0.01, compared with healthy subject groups. ^Δ^*P* < 0.05, compared with male group.

º*P* < 0.05, compared with wild-type AA homozygous genotypes.

**Appendix Table S10 Table S10**

**~~Appendix Table S10-1~~** ~~DNA methylation levels of global genome of focal adhesion Kindlin-2 (Fermt2) by genotyping for Exon 11 (CT, rs777658527) in Chinese healthy subjects (n=108) and Chinese hepatoma patients (n=130).~~

|  | SNP |  | Healthy subjects  (n= 108) | |  | |  | Hepatoma patients  (n=130) | | |  |  |
| --- | --- | --- | --- | --- | --- | --- | --- | --- | --- | --- | --- | --- |
| 14  (4-1) | Kindlin-2 (Fermt2)  rs777658527 |  | Frequencies | DNA metylation  of global genome | | *P* value | | | Frequencies | DNA metylation  of global genome | *P* value | *P* value |
|  | (Exon 11, C>T) |  | % (n) | (%) |  | |  | % (n) | | (%) |  |  |
|  | Genotype |  |  |  |  | |  |  | |  |  |  |
|  | wt/wt (CC) | 100.0 % (108/108) | | 1.30±0.303 |  | |  | 100.0 % (130/130) | | 1.518±0.656 ^# #^ | 0.0017 |  |
|  | wt/mu (CT) |  | 0.0 % (0) | 0.0±0.0 |  | |  | 0.0 % (0) | | 0.0±0.0 |  |  |
|  | mu/mu (TT) |  | 0.0 % (0) | 0.0±0.0 |  | |  | 0.0 % (0) | | 0.0±0.0 |  |  |
|  | mu carrier  (CT+ TT) |  | 0.0 % (0) | 0.0±0.0 |  | |  | 0.0 % (0) | | 0.0±0.0 |  |  |
|  | Allele |  |  |  |  | |  |  | |  |  |  |
|  | C | 100.0 % (216/216) | |  |  | |  | 100.0 % (260/260) | |  |  |  |
|  | T |  | 0.0 % (0) |  |  | |  | 0.0 % (0) | |  |  |  |
|  | Gender |  |  |  |  | |  |  | |  |  |  |
|  | Total |  | 108 | 1.30±0.303 |  | |  | 130 | | 1.518±0.656^# #^ | 0.0017 |  |
|  | Male |  | 45.4 % (49) | 1.294±0.303 |  | |  | 88.5 % (115) | | 1.570±0.647^# #^ | 0.0049 |  |
|  | Female |  | 54.6 % (59) | 1.304±0.305 |  | |  | 11.5 % (15) | | 1.118±0.609 ^Δ^ | 0.1153 | 0.0116 |

wt/wt (CC), Wild-type homozygous; wt/mu (CT), Variant heterozygous; mu/mu (TT), Variant homozygous; mu carrier (CT+TT), Variant allelic carrier. SNP: single nucleotide polymorphism.

^*^*P* < 0.05, ^#^*P* < 0.05, ^# #^*P* < 0.01, compared with healthy subject groups. ^Δ^*P* < 0.05, compared with male group.

**~~Appendix Table S10-2~~** ~~DNA methylation levels of global genome of focal adhesion Kindlin-2 (Fermt2) by genotyping for Exon 7 (GA, rs2357947) in Chinese healthy subjects (n=108) and Chinese hepatoma patients (n=130).~~

|  | SNP |  | Healthy subjects  (n= 108) | |  | |  | Hepatoma patients  (n=130) | | |  | |  |
| --- | --- | --- | --- | --- | --- | --- | --- | --- | --- | --- | --- | --- | --- |
| 15  (4-2) | Kindlin-2 (Fermt2)  rs2357947  (Exon 7, G>A) |  | Frequencies  % (n) | DNA metylation  of global genome  (%) | | *P* value | | | Frequencies  % (n) | DNA metylation  of global genome  (%) | | *P* value | *P* value |
|  | Genotype |  |  |  |  | |  |  | |  |  | |  |
|  | wt/wt (GG) |  | 10.2 % (11/108) | 1.209±0.154 |  | |  | 8.5 % (11/130) | | 1.636±0.643^#^ | 0.0447 | |  |
|  | wt/mu (GA) |  | 35.2 % (38/108) | 1.355±0.332 | 0.1659 | |  | 37.7 % (49/130) | | 1.625±0.63^#^ | 0.0188 | |  |
|  | mu/mu (AA) |  | 54.6 % (59/108) | 1.285±0.323 | 0.4493 | |  | 53.8 % (70/130) | | 1.427±0.671 | 0.1397 | | 0.0088 |
|  | mu carrier (GA+AA) |  | 89.8 % (97/108) | 1.313±0.327 | 0.3014 | |  | 91.5 % (119/130) | | 1.507±0.659 ^# #^ |  | |  |
|  | Allele |  |  |  |  | |  |  | |  |  | |  |
|  | G |  | 27.8 % (60/216) |  |  | |  | 27.3 % (71/260) | |  |  | |  |
|  | A | 72.2 % (156/216) | |  |  | |  | 72.7 % (189/260) | |  |  | |  |
|  | Gender |  |  |  |  | |  |  | |  |  | |  |
|  | Total |  | 108 | 1.302±0.315 |  | |  | 130 | | 1.518±0.656^# #^ | 0.0019 | |  |
|  | Male |  | 45.4 % (49) | 1.307±0.322 |  | |  | 90.0 % (117) | | 1.571±0.658^# #^ | 0.0082 | |  |
|  | Female |  | 54.6 % (59) | 1.30±0.311 |  | |  | 10.0 % (13) | | 1.118±0.609 ^Δ^ | 0.0192 | |  |

wt/wt (GG), Wild-type homozygous; wt/mu (GA), Variant heterozygous; mu/mu (AA), Variant homozygous; mu carrier (GA+AA), Variant allelic carrier. SNP: single nucleotide polymorphism.

^*^*P* < 0.05, ^#^*P* < 0.05, ^# #^*P* < 0.01, compared with healthy subject groups. ^Δ^*P* < 0.05, compared with male group.

º*P* < 0.05, compared with wild-type GG homozygous genotypes.

**Appendix Table S10-3** DNA methylation levels of global genome of focal adhesion Kindlin-2 (Fermt2) by genotyping for Exon 4 (T>A/T>C/

~~T>G, rs62003529) in Chinese healthy subjects (n=108) and Chinese hepatoma patients (n=130).~~

|  | SNP |  | Healthy subjects  (n= 108) | |  | |  | Hepatoma patients  (n=130) | | |  | |  | |
| --- | --- | --- | --- | --- | --- | --- | --- | --- | --- | --- | --- | --- | --- | --- |
| 16  (4-3) | Kindlin-2 (Fermt2)  rs62003529 |  | Frequencies | DNA metylation  of global genome | | *P* value | | | Frequencies | DNA metylation  of global genome | | *P* value | | *P* value |
|  | (Exon 4, T>A/T>C/  T>G) |  | % (n) | (%) |  | |  | % (n) | | (%) |  | |  | |
|  | Genotype |  |  |  |  | |  |  | |  |  | |  | |
|  | wt/mu (TA/TC/TG) |  | 0.0 % (0) | 0.0±0.0 |  | |  | 0.0 % (0) | | 0.0±0.0 |  | |  | |
|  | mu/mu (AA/CC/GG) |  | 0.0 % (0) | 0.0±0.0 |  | |  | 0.0 % (0) | | 0.0±0.0 |  | |  | |
|  | mu carrier  (TA/TC/TG + AA/CC/GG) |  | 0.0 % (0) | 0.0±0.0 |  | |  | 0.0 % (0) | | 0.0±0.0 |  | |  | |
|  | Allele |  |  |  |  | |  |  | |  |  | |  | |
|  | T | 100.0 % (216/216) | |  |  | |  | 100.0 % (260/260) | |  |  | |  | |
|  | A/C/G |  | 0.0 % (0) |  |  | |  | 0.0 % (0) | |  |  | |  | |
|  | Gender |  |  |  |  | |  |  | |  |  | |  | |
|  | Total |  | 108 | 1.302±0.315 |  | |  | 130 | | 1.518±0.656 ^# #^ | 0.0019 | |  | |
|  | Male |  | 37 % (40) | 1.339±0.309 |  | |  | 88.5 % (115) | | 1.571±0.658 ^#^ | 0.0335 | |  | |
|  | Female |  | 63 % (68) | 1.280±0.318 |  | |  | 11.5 % (15) | | 1.118±0.609 ^Δ^ | 0.0127 | |  | |

wt/wt (TT), Wild-type homozygous; wt/mu (TA/TC/TG), Variant heterozygous; mu/mu (AA/CC/GG), Variant homozygous; mu carrier (TA/TC/TG + AA/CC/GG), Variant allelic carrier. SNP: single nucleotide polymorphism.

^*^*P* < 0.05, ^#^*P* < 0.05, ^# #^*P* < 0.01, compared with healthy subject groups. ^Δ^*P* < 0.05, compared with male group.

º*P* < 0.05, compared with wild-type TT homozygous genotypes.

**Appendix Table S11-1 - Table S11-1**

**Table S11-1** Primer sequence, polymerase chain reaction (PCR) analysis conditions for focal adhesions Kindlin-1 and Kindlin-2 genes in single nucleotide polymorphism (SNP) in Chinese healthy subjects (n=116) and hepatic carcinoma patients (n=241). (Total: n=357).

|  | Gene name | Gene (SNP) | Reference SNP | Localization | Alternative names |
| --- | --- | --- | --- | --- | --- |
| 1 | Kindlin-1 (Fermt1) | Fermt1(Kindlin-1) | rs202037230 | Exon 7 | C>T |
| 2 | Kindlin-1 (Fermt1) | Fermt1(Kindlin-1) | rs2232074 | Exon 11 | C>T |
| 3 | Kindlin-1 (Fermt1) | Fermt1(Kindlin-1) | rs16991866 | Exon 3 | A>G |
| 4 | Kindlin-2 (Fermt2) | Fermt2(Kindlin-2) | rs777658527 | Exon 11 | C>T |
| 5 | Kindlin-2 (Fermt2) | Fermt2(Kindlin-2) | rs2357947 | Exon 7 | G>A |
| 6 | Kindlin-2 (Fermt2) | Fermt2(Kindlin-2) | rs62003529 | Exon 4 | T>A / T>C / T>G |

SNP: single nucleotide polymorphism. Kindlin-1 (Fermt1); Kindlin-2 (Fermt2).

**Appendix Table S11-2 - Table S11-2**

**Table S11-2** Primer sequence, polymerase chain reaction (PCR) analysis conditions for genotyping of focal adhesions Kindlin-1 (Fermt1) and Kindlin-2 (Fermt2) genes of single nucleotide polymorphism (SNP) in Chinese healthy subjects (n=116) and hepatic carcinoma patients (n=241).

|  | | Gene (SNP) | Reference SNP | Primer sequences | PCR product (bp) |
| --- | --- | --- | --- | --- | --- |
|  | 1 | Kindlin-1 (Fermt1)  C>T, Exon 7 | rs202037230 | Forward:5’-ACGTTGGATGCCTCATTACACACACACCTC-3’  Reverse:5’-ACGTTGGATGTGGCTTGCTCATAGAGTTGG-3’ | 95 |
|  | 2 | Kindlin-1 (Fermt1)  C>T, Exon 11 | rs2232074 | Forward: 5’-ACGTTGGATGAATGTTTTGTGTCACCACGG-3’  Reverse: 5’-ACGTTGGATGCTGCAAACAATTGCCCTAAC-3’ | 101 |
|  | 3 | Kindlin-1 (Fermt1)  A>G, Exon 3 | rs16991866 | Forward: 5’-ACGTTGGATGGGAGAACTCTCCAGGTTTAG -3’  Reverse: 5’-ACGTTGGATGCTTGTTAAAGCCGTCTGGTG-3’ | 122 |
|  | 4 | Kindlin-2 (Fermt2)  C>T, Exon 11 | rs777658527 | Forward: 5’-ACGTTGGATGTAAGGATGACTGGTCAACTC-3’  Reverse: 5’-ACGTTGGATGTGATTCCAGTTGCAGAAGGC-3’ | 131 |
|  | 5 | Kindlin-2 (Fermt2)  G>A, Exon 7 | rs2357947 | Forward: 5’-ACGTTGGATGATCTCTTCCAGGAGAATGGC-3’  Reverse: 5’-ACGTTGGATGTGTTGCAGTATGATGCAATC-3’ | 93 |
|  | 6 | Kindlin-2 (Fermt2)  T>A / T>C / T>G,  Exon 4 | rs62003529 | Forward: 5’-ACGTTGGATGTGGCAAGGCTCACCTTTCAG-3’  Revrse: 5’-ACGTTGGATGGTTGTGGCCTTTCCTTTACC-3’ | 116 |

SNP: single nucleotide polymorphism. Kindlin-1 (Fermt1); Kindlin-2 (Fermt2).

**Appendix Table S12- Table S12**

**Appendix Table S12** Primers, conditions and products of PCR amplification, and cytosine residue rates of bisulfite conversion in promoter region of Kindlin-2 gene.

|  | Amplicon Name | Left  Primer | Right Primer | **Direction** | LPL | RPL | Target Sequence | Taregt Length | Target CpG | CpG Analyzed InT | Left Primer Plus Tag | Right Primer  Plus Tag |
| --- | --- | --- | --- | --- | --- | --- | --- | --- | --- | --- | --- | --- |
| 3 | Kindlin-2-8  **(3-1-0-8)** | GGAGAGGGGTTATAGGTTTTTG | AACCAAAAAAATCCCCAACT | F | 22 | 20 | GGAGAGGGGCCACAGGCTCCTGGCCTTTCTAAGCACACCAAGTGCCCAGTCGCGGACCCCCGGGACCAGGATGCGCTGACGACCCGGCTGGCAGGCGGGTCCTCGTGGGCGAGGCGAGGGAGGCGGCGAGAGAGGAGCAATAGTTTCCCACCGCTCCCTCTCAGGCGCAGGGTCTAGAGAAGCGCGAGGGGATCTAGAGAAGCCGGAGGGGAGGAAGCGCGAGTCCGCGGCCCGCCCCGTTGCGTCCCACCCACCGCGTCCCCTCCCCTCCCCTCCCGCTGCGGGAAAAGCGGCCGCGGGCGGCGGCGCCCACTGTGGGGCGGGCGGAGCGCCGCGGGAGGCGGACGAGATGCGAGCGCGGCCGCGGCCCCGGCCGCTCTGGGCGACTGTGCTGGCGCTGGGGGCGCTGGCGGGCGTTGGCGTAGGAGGTGAGTGAGGCTCCGGCTCGGCAGCGTCGCAGCTGCCCCAGGATCTGCGCCCCGGTCAAGTTGCGGACTTGGAGCCGGCAAACGCGGAGGGCTGGTCCCGCGCGTCTGCGCTGGGAATGCGCGTGTCCTGGCTGGCGCGGTCGGAGCCGGGAGCTGGGGACCTTCCTGGCC | 599 | 74 | 36 | aggaagagagGGAGAGGGGTTATAGGTTTTTG | cagtaatacgactcactatagggagaaggctAACCAAAAAAATCCCCAACT |
|  |  |  |  |  |  |  |  |  |  |  |  |  |
|  | Kindlin-2-13  **(3-2-0-13)** | TTTAGAAAGGTTAGGAGTTTGTGGTT | TTTCAAACACTAACTAAATACAAACAAA | R | 26 | 28 | CTTAGAAAGGCCAGGAGCCTGTGGCCCCTCTCCGGACCCTTCCAGTCCCGCCTCCTGGGAGACCCTGTTTCGCTGGATTCTTGGGACACATTCACCCACTACCTCGAGTTTCATTCATTCACACTTTAATTCATTTGACTGGCAAACATCTGCTGAATGCCTTCTCTGAACCGGTTCAGTCCTTTATTATTCACTAATTTTTTTCTTGCCTTAGTTTGGTTTTCCGGTAGACTACCTACCTGTTGAGGGCAAGACCACATCCCAAGCTTGCGTAAGCAGTGATAGGTCCATGAAGAGCCTCGCCCTACAAACACTTGCTTGCACCTAGCCAGTGTCTGAAA | 341 | 8 | 8 | aggaagagagTTTAGAAAGGTTAGGAGTTTGTGGTT | cagtaatacgactcactatagggagaaggctTTTCAAACACTAACTAAATACAAACAAA |

Kindlin-2: focal adhesion~~s~~ family member2, integrin tail binding protein Kindlin-1(Fermt1), Fermt2: fermitin family member 2；

CpG: cytidine-phosphate-guanosine dinucleotides,

Bp: base pairs. TSS: Transcription Starting Site. F: Forward; R: Reverse. LPL: Lower Primer Length.

**Appendix Figure S1 Figure S1**

**Kindlin-2 promotor sequence (Range from-1bp /-481 bp)**

Kindlin-2 promoter sequence (-1bp / -481 bp)

-481 aattagtgaa taataaagga ctgaac**cg**gt tcagagaagg cattcagcag atgtttgcca

32

-421 gtcaaatgaa ttaaagtgtg aatgaatgaa act**cg**aggta gtgggtgaat gtgtcccaag

31

-361 aatccag**cg**a aacagggtct cccaggagg**c g**ggactggaa gggtc**cg**gag aggggccaca

30 29 28

-301 ggctcctggc ctttctaagc acac caagtg cccagt**cgcg** gacccc**cg**gg accaggatg**c**

27 26

-241 **g**ctga**cg**acc **cg**gctggcag g**cg**ggtcct**c g**tggg**cg**agg **cg**agggagg**c g**g**cg**agagag

25 24 23 22 21 20 19 18

-181 gagcaatagt ttcccac**cg**c tccctctcag g**cg**cagggtc tagagaag**cg cg**aggggatc

17 16 15

-121 tagagaagc**c g**gaggggagg aag**cgcg**agt c**cgcg**gcc**cg** ccc**cg**ttg**cg** tcccacccac

14 13 12 11 10 9

-61 **cgcg**tcccct cccctcccct cc**cg**ctg**cg**g gaaaag**cg**gc **cgcg**gg**cg**g**c g**g**cg**cccact

8 7 6 5 4 3 2 1

-3001 ttttcaaaag tgtcaaagtg atgaatgaga aagactgaga aaatgtcaca gattgaggcc

-2941 aactaaggag acatgatgac taaatacaat ggattagatc ctggaccaga aaaagaacat

-2881 taatgggaaa aaaaatggta aaattcaaat tctgtagcta gatgaattga tagtattata

-2821 t**cg**atgttaa tttcctgtgt ttgataatta tgatgtagaa atgtaagatg tgaa**cg**ttag

-2761 gggaagctgg gtgaaggaca taagaaaact ctctttagta ttttgtaact cttctgtaag

-2701 tattaatgat ttcaaaataa aaagttaggg aaaaaaatcc aggaggagaa gaaagagaaa

-2641 caagaatata aaggatggca gacttctcac cagagatgat ggagacagca aacagcagac

- 2581 catctttaat gtacttgaag agacaagtct caatcaattt agaagtttat ttggccaagg

-2521 ttaagcacat gcccatgaca tagcctcagg aagtcctgaa gaca**cg**tgcc caaggtggtc

-2461 aggctacagc tcagttttat acattttagg gagacaaaag acatcaatca atacatgtac

-2401 attgaacatt ggttcagtcc agaaaggcag gacatctgac acagggactt ccaggttaga

-2341 ga**cg**gattca aagatttttt cattggcaat tggttgtaag tgttattatc taaagaccta

-2281 gaaccaacag aaaggaatgt ctgagttagg ataaagggtt gtggagacca aacttttatc

-2221 atgcagatga agccagaggc ttcagacaga atagactgtt aatgtttctt atcagactta

-2161 gagagtctat tctatcagcc ttaaggtctt tgtgttgatg ctaatgctgg tcagctgtga

-2101 ggcatattca accaccaccc cccctccacc ttccccaaac ctgcttcaca tcatggtctg

-2041 aactagtttt tcagattaac tttggaatgc ccttggctga gaggaagggt tagttcagac

-1981 agttgtgggg gggcttataa tgttattttt agtttacagg ttcttacaat ttacatagaa

-1921 ttgttcaata ttaattcaac ataggctact gtatgataag ttaaggagga atattgtaat

-1861 acctagagca aacacacaca cacacacaca cacacacaca cacacatgca aa**cg**aggtgt

-1801 agctaggaag ccagtagaga aaaaaaaatt atcccaaaag aaagcagaaa aagagaaaca

-1741 gagaaaccag ccaaaaagat agaa**cg**aata acaagatagt aaatgtaaac acaaccatca

-1681 attactatac taaatgtgaa tggactagct cctttgcctt ccctggggct gggagtgcag

-1621 gatggactca cttgcacaat tatgtcatgt ttgctagggc ttgggctagg gttgtggact

-1561 tagcatactt gctgaggcca gtgcccaccc caagtggttg ctgacagcag ggataggcta

-1501 aggagacacc taagtggctg gagattggtt ttatatatat gggaatggag cagataacta

-1441 aatacttagg cagagatgta accaggccat gtgacctgaa ctttgctcca gggcattgac

-1381 actgctgtta aaatgcttct ggcctagagg ccctctgcag ccaggggagg tagatggagc

-1321 ttcagcctga aaacccaggt cagagggccc aagtaccctt tgcagagctg gcttttcccc

-1261 ttgcaaattg ctagtga**cg**c ttcagctgat gtgtgttact attaaggtcc tagtgtttgg

-1201 gagggtgggg caggaggtgg aggattgtca gaaaaaaatt acatggaaaa agatggcatc

-1141 tgagatgttt tgaaagataa gtggaatttt ccaagtggaa aaaggaagga aaatcagtca

-1081 gataggaagg catggagcat tggggaatga caagtatctt cttggactag ggtgggagat

-1021 gggctggaga gatgggtcag ggccagttgt ctggcatctt gtgtgtctca gaagagggtg

-961 ggca**cg**ctg**c g**tagggaagc ccagggccac tctgaaagcc ctaaagggga actgatgcct

-901 ctggccttgt ttttatcacc atcaggacta cccattgagg caggctgcac taccagctac

-841 ttcctggtgc cctcttgctc atagccatag tattttgcct ctctgagctt ccagaggttt

-781 taagtctggg gaagacccag ggactcaaag aaagattggg gtgggagata aggggccaca

-721 gtttggggga gtcaggcagg aggcctttga ggaaaataga taaagtccca aagcctgtga

-661 gtgtgaattt ggaggcaata tgctgtgttc tgaaa**cg**ttt tcagacactg gctaggtgca

-601 agcaagtgtt tgtaggg**cg**a ggctcttcat ggacctatca ctgctta**cg**c aagcttggga

-541 tgtggtcttg ccctcaacag gtaggtagtc tac**cg**gaaaa ccaaactaag gcaagaaaaa

-481 aattagtgaa taataaagga ctgaac**cg**gt tcagagaagg cattcagcag atgtttgcca

-421 gtcaaatgaa ttaaagtgtg aatgaatgaa act**cg**aggta gtgggtgaat gtgtcccaag

-361 aatccag**cg**a aacagggtct cccaggagg**c g**ggactggaa gggtc**cg**gag aggggccaca

-301 ggctcctggc ctttctaagc acaccaagtg cccagt**cgcg** gacccc**cg**gg accaggatgc

-241 gctga**cg**acc **cg**gctggcag g**cg**ggtcct**c g**tggg**cg**agg **cg**agggagg**c g**g**cg**agagag

-181 gagcaatagt ttcccac**cg**c tccctctcag g**cg**cagggtc tagagaag**cg cg**aggggatc

-121 tagagaagc**c g**gaggggagg aag**cgcg**agt c**cgcg**gcc**cg** ccc**cg**ttg**cg** tcccacccac

-61 **cgcg**tcccct cccctcccct cc**cg**ctg**cg**g gaaaag**cg**gc **cgcg**gg**cg**g**c g**g**cg**cccact

-1 g

-1

From transcription start site (TSS, -1) upstream sequence (promotor region),

To transcription start site (TSS, +1) downstream sequence (coding region)

+1

**1** gtgggg**cg**gg **cg**gag**cg**c**cg** **cg**ggagg**cg**g a**cg**agatg**cg** ag**cgcg**gc**cg** **cg**gccc**cg**gc

61 **cg**ctctggg**c g**actgtgctg g**cg**ctggggg **cg**ctgg**cg**gg **cg**ttgg**cg**ta ggaggtgagt

121 gaggctc**cg**g ct**cg**gcag**cg** t**cg**cagctgc cccaggatct g**cg**ccc**cg**gt caagttg**cg**g

181 acttggagc**c g**gcaaa**cgcg** gagggctggt cc**cgcgcg**tc tg**cg**ctggga atg**cgcg**tgt

241 cctggctgg**c gcg**gt**cg**gag c**cg**ggagctg gggaccttcc tggcc**cg**g**cg** gtgg**cg**gggc

301 tgagaggtgc ctggtgcctg ggag**cg**ggtc ctc**cg**aga**cg** c*cg*aagccat cagccaggct

361 gag**cg**ccttc c**cg**gc**cg**c**cg** **cg**g**cgcg**ca**c g**gtg**cg**gtgg g**cg**catctct gagccc**cgcg**

421 ctcacc**cg**gg gctg**cgcgcg** t**cgcg**ggtgg tcctggt**cg**g gccaagga**cg** actggcccag

481 gagccaagg**c g**aggtggggc ttc**cg**ggggt tgttcc**cgcg** ccttggcaga gggatgc**cg**c

541 tgtagcttcc tgggtgagag **cg**tg**cgcgcg cgcg**ggtttg ccagtttccc cacatttcca

601 atttctcctg tta**cg**ctttc tccagaaggt tttttctttt ccttttttct ttctttcttt

661 ctttttttta ccttcaactt ggcttcctg**c g**gggttgttt tggagcagga tgagggcttt

721 gcctcctcca gtgtccccag gtggcag**cg**g tgcctctgct cccagggcag accctgcacc

781 **cg**aaaaatct ctagtgtatt **cg**gggagcca tgaaaaggct cccttgggc**c g**gtgggatcc

841 ttggctttgt ctctggctgc tg**cg**cacac**c g**tcagc**cg**tc agggcaattg gcatt**cg**gcc

901 tctttggtac tggggacaca ggtggaggga tggag**cg**ggc acagcc**cg**gg gttgctgccc

961 gtggtcctct ctgagtcctt ggtgattttg cctgggctgg agagaaaccc tggctcctgg

1021 tctgctgggg c**cg**cctctgc ctcagaggat gacacaagag tgcatgtaag tatttttaat

1081 aaaaactgta gtact**cg**taa aacaatctac accctgcaga agggatttgt tattttattt

1141 tattatttat ttatttattt atttattttt gaga**cg**gagt cttgctctgt **cg**cccaggct

1201 ggagtgcagt ggtgggttct **cg**gctcactg caacttctgc ctcctgggtt taag**cg**attc

1261 ttctggctca gcctcc**cg**ag tagctgggat tacagg**cg**cc tgccaccacg gc**cg**gctaat

1321 ttttgtattt ttagtagaga **cgcg**gtttca ccatgttggc caggctggtc tggagctcct

1381 ggcctcaagt gatc**cg**ccca cctcagcctc ccaaagtgct gggaatacag g**cg**tgagcca

1441 c**cg**cacctgg ccaggattta ttttaaaaag ggaagatttg ttgataaatt cacttcaaag

1501 ataaactatt **cg**aaaatact ttagtgattc c**cg**tcaagac tcttctgtgt atgtatagac

1561 gtataactca ttctggacag ggcaaggata tctttttttg tttgtttgtt tgttttgaga

1621 tggactct**cg** ctgt**cg**ccag gctagagtgc agtgg**cgcg**a tttcagctca ctgcaacctc

1681 **cg**cttcc**cg**g gttcaaggga ttctcctgcc tcagcctcc**c g**agtagctgg gattacaggc

1741 a**cg**caccacc a**cg**ccctact aatttttgta tttttagtag aga**cg**ggatt tccccatgtt

1801 ggccaggatg atct**cg**atct cttgaccc**cg** tgatcagcct gccttggcct cccaaagtgc

1861 tgggattaca gg**cg**tgagcc ac**cg**cacc**cg** gccaagggta tcttgaagga gggattacag

1921 ttgatatgta gaggaatatt gcagtggtta ttgctgcatt tcctatgtga ctgggactaa

1981 aacagatcag ctgatagtgt tag**cg**tgcag tgagcagtct gatgactatg acacagaaat

2041 aagaatctcc agcattctgc cctgggacat gtggagcctt gggtcagatg ctgctggcta

2101 ttgatgc**cg**g gaatggagaa atccaccagg gcaactcagt aaaaataaat aaataaataa

2161 ataaataaat acataattaa aaaaagggta tccttgtcct gtccaggatg agatgcttgc

2221 ttccatgcac tta**cg**acaat ttttgctgtc attaaaaaat tttcacattc acagtcttct

2281 aaattcactt tggagtgtat tggattccac tgcattgaca ta**cg**taagtt tgcattaaaa

2341 gatcctttaa gatatctggt caggca**cg**gt ggctca**cg**cc tgtaatccca gcactttggg

2401 aagtggaggt agaaggattg cttgagccca ggagtt**cg**ag accagcttgg gcaacatagt

**Appendix Figure S1 Legend**

**Figure S1 Schematic diagram of focal adhesion Kindlin-2 gene proximal promoter sequence from -1 bp to -481 bp and location of thirty-two interspersed cytidine-phosphate-guanosine dinucleotides (CpG) sites.**

The sequence shown represents a fragment from -1 base pairs (bp) to -481 bp in the 5’ untranslated region (5’-UTR) of promoter. CpG sites were numbered sequentially in these regions separately, and underlining denotes the CpG units containing more than one CpG site detected all together. Polymerase chain reaction primers were designed on the basis of the reverse complemented strand of this fragment.

(2-3)

（Kindlin-2-8-(jpeg)—24 samples）


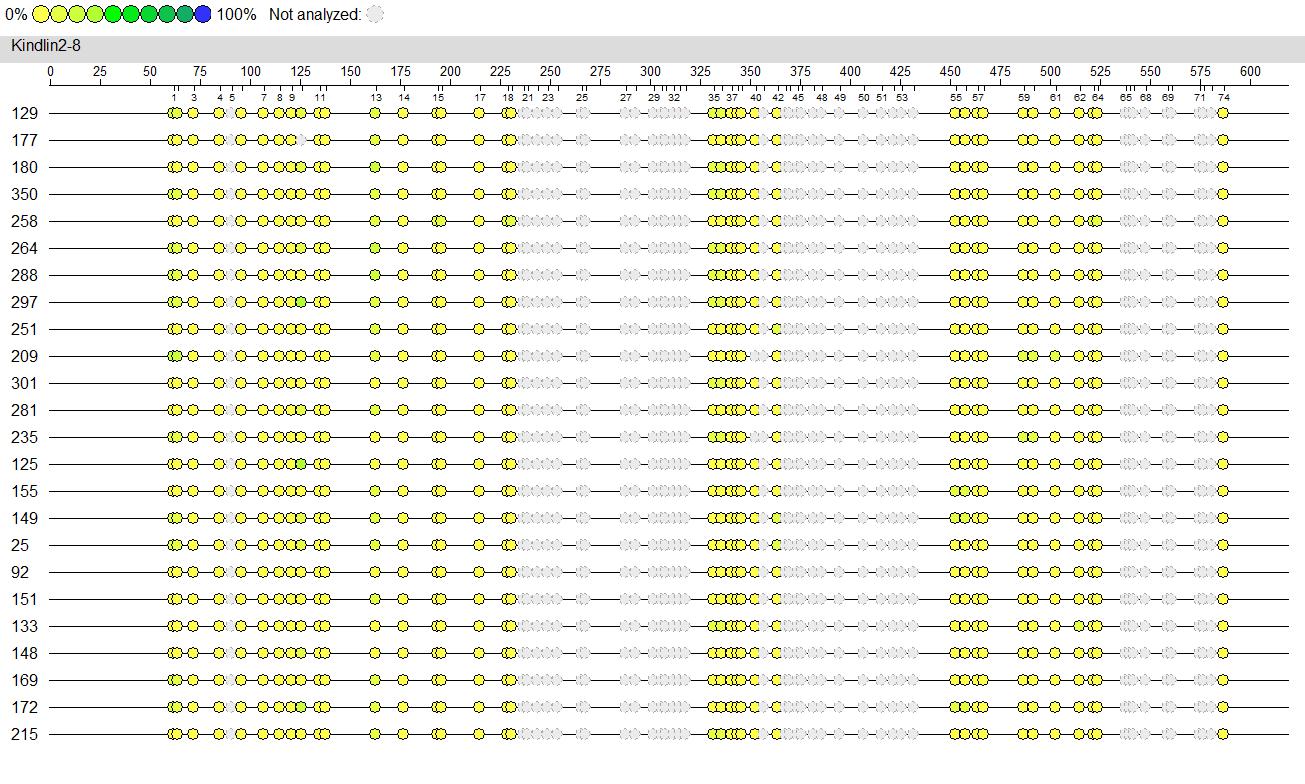


| **1** |
| --- |
| **2** |
| **3** |
| **4** |
| **5** |
| **6** |
| **7** |
| **8** |
| **9** |
| **10** |
| **11** |
| **12** |
| **13** |
| **14** |
| **15** |
| **16** |
| **17** |
| **18** |
| **19** |
| **20** |
| **21** |
| **22** |
| **23** |
| **24** |
